# Immunity priming and biostimulation by airborne nonanal increase yield of field-grown common bean plants

**DOI:** 10.3389/fpls.2024.1451864

**Published:** 2024-11-05

**Authors:** Iris J. Elizarraraz-Martínez, Mariana A. Rojas-Raya, Ana A. Feregrino-Pérez, Laila P. Partida-Martínez, Martin Heil

**Affiliations:** ^1^ Departamento de Ingeniería Genética, Laboratorio de Ecología de Plantas, Centro de Investigación y de Estudios Avanzados (CINVESTAV) - Unidad Irapuato, Irapuato, Mexico; ^2^ Facultad de Ingeniería, Universidad Autónoma de Querétaro, Querétaro, Mexico; ^3^ Departamento de Ingeniería Genética, Laboratorio de Interacciones Microbianas, Centro de Investigación y de Estudios Avanzados (CINVESTAV)- Unidad Irapuato, Irapuato, Mexico

**Keywords:** biological control, crop protection, maternal effects, *Phaseolus vulgaris*, transgenerational resistance, translational research, volatile organic compound (VOC)

## Abstract

**Introduction:**

Stress-induced volatile organic compounds (VOCs) that induce plant immunity bear potential for biocontrol. Here, we explore the potential of nonanal to enhance the seed yield of common bean (*Phaseolus vulgaris*) under open field conditions that are realistic for smallholder farmers.

**Methods and results:**

Using plastic cups with a nonanal-containing lanolin paste as low-cost dispensers, we observed that exposure of Flor de Junio Marcela (FJM) plants over 48h to airborne nonanal was followed by a 3-fold higher expression of pathogenesis-related (PR) genes PR1 and PR4. Both genes further increased their expression in response to subsequent challenge with the fungal pathogen *Colletotrichum lindemuthianum*. Therefore, we conclude that nonanal causes resistance gene priming. This effect was associated with ca. 2.5-fold lower infection rates and a 2-fold higher seed yield. Offspring of nonanal-exposed FJM plants exhibited a 10% higher emergence rate and a priming of PR1- and PR4-expression, which was associated with decreased infection by *C. lindemuthianum* and, ultimately, a ca. 3-fold increase in seed yield by anthracnose-infected offspring of nonanal-exposed plants. Seeds of nonanal-exposed and of challenged plants contained significantly more phenolic compounds (increase by ca 40%) and increased antioxidant and radical scavenging activity. Comparative studies including five widely used bean cultivars revealed 2-fold to 3-fold higher seed yield for nonanal-exposed plants. Finally, a cost-benefit analysis indicated a potential economic net profit of nonanal exposure for some, but not all cultivars.

**Outlook:**

We consider nonanal as a promising candidate for an affordable tool that allows low-income smallholder farmers to increase the yield of an important staple-crop without using pesticides

## 1 Introduction

Plants respond to abiotic and biotic stress with the induced emission of volatile organic compounds (VOCs), which contribute via diverse mechanisms to the protection from future stresses. Since the pioneering work of Marcel Dicke (Dicke, 1986; 1988; Dicke and Sabelis, 1988) and Ted Turlings (Turlings et al., 1990, 1995), tritrophic interactions in which herbivore-induced plant VOCs guide predatory mites or parasitoid wasps towards their prey have been studied intensively, in the hope to translate this plants’ ‘cry for help’ into novel biocontrol strategies. Even earlier, David Rhoades (Rhoades, 1983) and Ian Baldwin & Jack Schultz (Baldwin and Schultz, 1983) published the first reports on talking trees. Although the idea of an airborne plant-plant communication initially received heavy criticism, multiple follow-up studies confirmed that VOCs from damaged plants can trigger increased immunity to biotic stress in a receiver plant (Heil and Silva Bueno, 2007; Yi et al., 2009; Heil, 2014; Rosenkranz et al., 2021; Brambilla et al., 2022; Schuman, 2023). Unfortunately, attempts to apply this knowledge in agricultural settings had limited success (Brilli et al., 2019; Schuman, 2023). For example, exposure of maize or cotton plants to methyl jasmonate (MeJA) under open field conditions induced defence traits as expected, but had no significant effect on yield (von Merey et al., 2012; Williams et al., 2017). Likewise, the emission of an aphid alarm pheromone by transgenic wheat plants – a reliable attractant of parasitoids under laboratory conditions - had no effect on seed yield in the open field (Bruce et al., 2015). Exposing common bean in experimental field plots to methyl salicylate (MeSA) had a significant effect on seed yield in one, but not another year (Salamanca et al., 2018), and the exposure of lima bean and chilli pepper plants in a common garden to *cis*-hexenyl acetate slightly increased the number of flowers in lima bean, but decreased the number of fruits in chili pepper (Freundlich et al., 2021). In short, with a few exceptions (see, e.g (Shiojiri et al., 2021), or the so-called push-pull system (Khan et al., 2014)), field studies tend to fail to reproduce the promising observations made under controlled conditions.

More reliable effects could be expected from VOCs that (i) act via more than one mechanism against the same enemy or (ii) exert more than one positive effect for the crop plant. For example, the C9 aliphatic aldehyde, nonanal, primes resistance gene expression and thereby enhances the resistance to bacterial and fungal pathogens (Yi et al., 2009; Girón-Calva et al., 2012; Quintana-Rodriguez et al., 2015), but nonanal has also direct antimicrobial activity (Bisignano et al., 2001; Dilantha-Fernando et al., 2005; Zhang et al., 2017; Quintana Rodríguez et al., 2018b; Li et al., 2021). Likewise, many biostimulants enhance plant growth and the resistance to stress (Mannino, 2023). Unfortunately, growth promotion, a well-known feature of microbial VOCs such as acetoin, 2,3-butanediol or nonan-2-one (Ryu et al., 2003; Sharifi and Ryu, 2018; Lammers et al., 2022; Almeida et al., 2023), has less frequently been reported for plant VOCs (Brosset and Blande, 2022). Finally, stable, long-term effects would be excepted from (iii) those VOCs that generate an immunological memory, that is, they prime the receiver plant for a faster or stronger responses to future attack, including the enhanced emission of VOCs (Heil and Silva Bueno, 2007; Ton et al., 2007; Erb et al., 2015; Quintana-Rodriguez et al., 2015; Brambilla et al., 2022).

The before mentioned studies call for a shift towards considering resistance-inducing and antimicrobial VOCs more seriously as tools for biocontrol. However, field studies are rare and in fact, a recent quantitative review identified the dominance of reports from ‘more refined, controlled environments’ as the major limitation of the literature on plant volatiles (Schuman, 2023). In addition, yield is seldomly quantified, and even in field studies, treatment costs - and in particular the ratio between these costs and the economic value of a putative yield increase - are usually not reported. These conditions and response variables hardly ever meet the reality in agriculture, and less so the reality under which smallholder farmers operate: a working force of around 600 million farmers around the world who are estimated to produce – on less than two hectares of land per farmer - more than 30% of the world food supply (Ricciardi et al., 2018; Shroff, 2022). For these farmers, variation in the abiotic and biotic conditions form an intrinsic element of their all-day reality that contributes significantly to a high level of food insecurity, and positive income/investment ratios are essential for survival.

Considering the before mentioned issues, we aimed to explore the potential of nonanal, to be applied as a biostimulant that increases seed yield of common bean under growing conditions that are realistic for Mexican smallholder farmers. Nonanal was identified by our group as a VOC emitted from infected lima bean or common bean plants that primes the expression of ‘pathogenesis- related’ (PR) genes PR1 and PR4 and thereby enhances the resistance to *Pseudomonas syringae* and *Colletotrichum lindemuthianum*, at least under laboratory conditions (Yi et al., 2009; Girón-Calva et al., 2012; Quintana-Rodriguez et al., 2015). Others reported that common bean emits nonanal in response to colonization with mycorrhizal fungi or sider mite infestation (Schausberger et al., 2012). We selected nonanal because it is a product of the lipoxygenase pathway, but also of the non-enzymatic oxidation of fatty acids (Fries, 1960), for which reason nonanal emission is usually induced by oxidative stress or infection (Wildt et al., 2003). Being an indicator of oxidative stress, nonanal can be considered a volatile damage-associated molecular pattern (DAMP): an endogenous molecule that indicates danger when it appears in the extracellular space (Heil and Land, 2014; Vénéreau et al., 2015; Tanaka and Heil, 2021). In fact, nonanal is also a common component of the breath of human cancer patients (Fuchs et al., 2010; Callol-Sanchez et al., 2017). Since DAMPs are the first alarm signals that are released in wounded tissue, they usually have antimicrobial, immunostimulatory and pro-regenerative activity (Medina-Castellanos et al., 2018; Segonzac and Monaghan, 2019; Gong et al., 2020). We argue that nonanal might have the same effects in plants. Finally - and importantly for the aim of our study - nonanal is relatively cheap (1kg less ca. 3,000 Mexican Pesos (MXN)), and it is widely used in washing & cleaning products, fragrances, cosmetics and other personal care products, because it is classified under FDA UNII code 2L2WBY9K6T and CAS no.: 124-19-6 as an organic substance of natural origin that is permitted as food additive and for direct human consumption (CHEM. E, 2023; FDA, 2023). That is, in case of having beneficial properties in the agricultural context, its certification for use as a biocontrol agent should be relatively easy.

Our study consisted of two trials: In the first trial, we challenged nonanal-exposed plants of the cultivar Flor de Juno Marcela with *C. lindemuthianum*, the causal agent of fungal anthracnose, to verify whether the priming of PR-genes by nonanal is reproducible under field conditions and whether resistance induction translates into increased seed yield. We also tested for putative transgenerational resistance priming, because the generation of a stable immunological memory in the next generation could amplify the opportunities to use VOCs as ‘crop vaccines’ (Ramirez-Carrasco et al., 2017), and we used standard protocols to estimate the contents of phenolic compounds and the antioxidant activity of the harvested beans to screen for changes in compound classes that contribute to the nutraceutical effects of beans. In a second trial, we compared the effects of nonanal exposure among five bean cultivars. We observed yield increases in response to nonanal exposure. In most cultivars, these yield increases could have generated a net economic profit. We could also confirm a transgenerational resistance priming. We conclude that nonanal is a very promising candidate for applications as a biostimulant that can be used by smallholder farmers to obtain yield increases at affordable costs while fulfilling the requirements of organic farming. In addition, seed-producing companies might use nonanal to generate ‘vaccinated’ seeds as a new product.

## 2 Materials and methods

### 2.1 Biological material and growing conditions

We used three improved cultivars and two landraces of common bean (*Phaseolus vulgaris* L.), which had been selected to cover a range of resistance levels to fungal anthracnose and to include cultivars that are commonly used for autoconsumption (Borja Bravo et al., 2021): The improved cultivars Flor de Mayo Anita (FMA) and Pinto Villa (PV) have been characterized as ‘highly resistant’, while the improved cultivar Flor de Junio Marcela (FJM) and the landrace Bayo Berrendo (BaB) are considered as ‘intermediate resistant’ (Acosta-Gallegos et al., 1995; Castellanos-Ramos et al., 2003; Ibarra-Perez et al., 2005). A previous own study confirmed these patterns and identified the landrace Negro San Luis (NSL) as highly susceptible (Quintana-Rodriguez et al., 2015). Flor de Mayo and Flor de Junio beans are cultivars destinated to autoconsumption, and also NLS is frequently used for this purpose (Borja Bravo et al., 2021). The seeds of BaB were obtained from the germplasm collection at Instituto Tecnológico de Roque (ITR), all other cultivars were obtained from Dr. Jorge Acosta at Instituto Nacional de Investigaciones Forestales, Agrícolas y Pecuarias (INIFAP), both in Celaya, GTO, México. The fungal pathogen *C. lindemuthianum* (Sacc. & Magnus) Briosi & Cavara strain 1088, which had been isolated originally from leaves and pods of naturally infected bean plants in the state of Durango, México, was kindly donated by Dr. June Simpson (CINVESTAV Irapuato) and was cultivated in Petri dishes with solid potato dextrose agar (PDA) medium.

All experiments were carried in an experimental field at CINVESTAV Campus Irapuato (state of Guanajuato, 20° 43′ 13″ N; 101° 19′ 43″ W). The study site is localized in the ‘Bajio’, a semiarid region in the central highlands of Mexico at ca 1,800 m asl. that receives an annual rainfall of ca 400 mm (Castellanos-Ramos et al., 2003). In this region of México, bean is typically cultivated during the rainy season (from March until August) under rainfed conditions (in entire Mexico, 76% of the bean production is rainfed (SAGARPA, 2017)). Some farmers also use supplemental irrigation at very different levels of control and intensity. Fungal, bacterial and (to a lesser extent) viral infections, pest insects, inefficient cultivation conditions and poor soils are the major yield-limiting factors in the Bajio, although higher peak temperatures and irregularities in rainfall increasingly impose major problems (Shulaev et al., 1997; Castellanos-Ramos et al., 2003). Therefore, average productivity has dropped to ca 800kg ha^-1^ (García-Meza, 2021), which is similar to the worldwide average of 790kg ha^-1^ (Uebersax et al., 2023). Small producers who grow bean for commercialisation typically sow two seeds into the same hole, in rows at 76 cm distance between rows, thereby reaching densities from 40,000 to 80,000 plants ha^-1^. Smallholder farmers who cultivate bean for autoconsumption on plots from a few up to 500 m^2^ will usually follow the same scheme, or simply seed a few rows with little distance between plants and irregular distances between rows (SAGARPA, 2017; Kushaha et al., 2024).

Since we aimed at mimicking these conditions, we grew plants from seed directly sown in the soil. Two seeds were sown into the same hole, in rows with 30 cm distance between plants and 60 cm distance between rows. Plants were watered manually two times per week until harvest, adding once per week a commercial fertilizer (FerViaFol 20:30:10) during the first two months. Weeding was also performed manually, and no pesticides were used. Our study consisted of two trials. In Trial 1 (transgenerational immunity) we investigated the effects of nonanal exposure on the expression of PR1 and PR4, resistance to *C. lindemuthianum*, and seed yield in the cultivar FJM, and to test for potential transgenerational effects. In Trial 2 (cultivar dependency) we compared the growth-and yield-enhancing effects of nonanal exposure among five cultivars.

### 2.2 Treatments

#### 2.2.1 Treatments of F0 plants (both trials)

**Figure 1:**
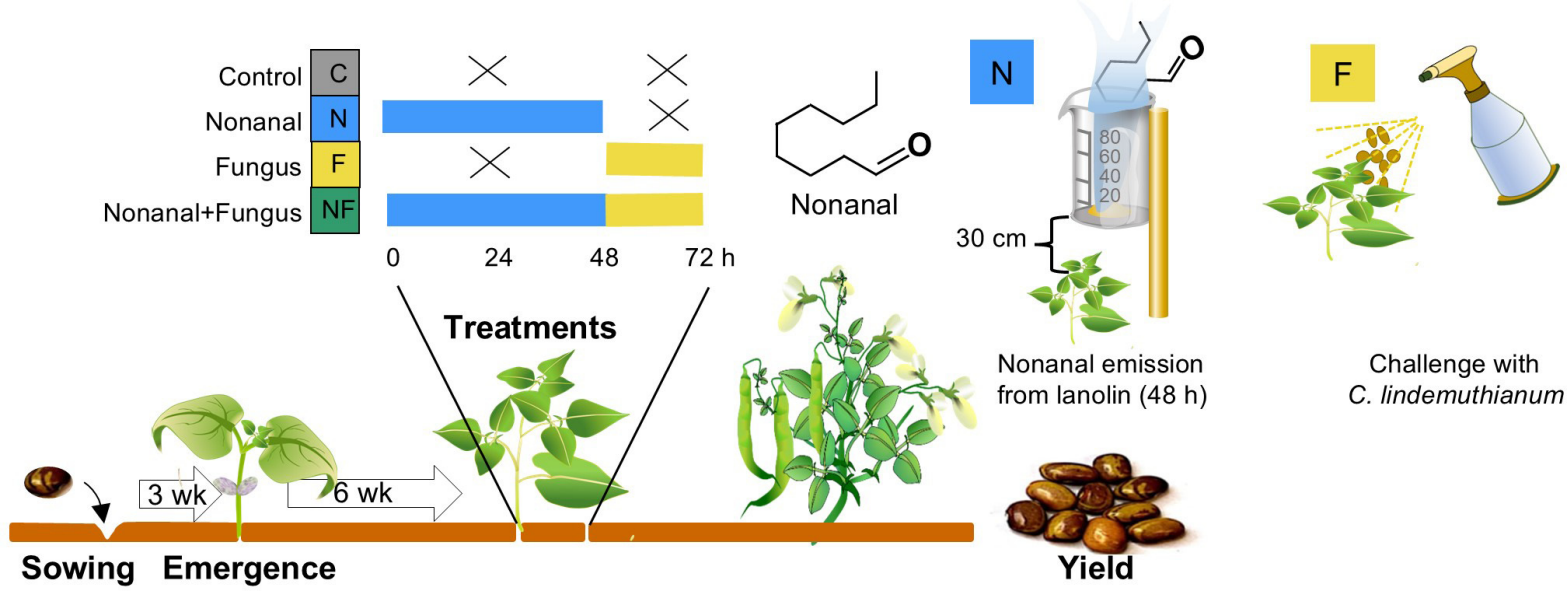
Experimental design, timing of treatments and sampling.

To study the effects of nonanal exposure on PR gene expression, anthracnose resistance and yield (Trial 1, March-April 2017), and to generate the seeds for the F1 generation, we established four plots of 4 x 6 m each that were separated by 3 m-wide margins of plant-free space and in each plot, we sew 200 FJM plants. We established similar plots for Trial 2 (August-December 2019), but in this case, we grew 25 plants of each of the five genotypes in each plot. At six weeks after emergence (at this moment, the plants had 3-5 trifoliate leaves), all plants in one plot received one of the following treatments (see **Figure 1** for a graphical overview of treatment and sampling schemes).

##### 2.2.1.1 Nonanal exposure

Exposure to nonanal over 48 h. To ensure that plants were only exposed to airborne nonanal, we used lanolin paste as a matrix from which nonanal was evaporated (Kost and Heil, 2006). Nonanal at analytical grade was dissolved in lanolin (both purchased from Sigma-Aldrich, St. Louis, Missouri, USA) at 8.1 mg g^-1^ (w/w nonanal in lanolin) in trial 1 and 12.4 mg g^-1^ in trial 2. These different concentrations were chosen to control for the different plot sizes. Two grams of this lanolin paste were collocated in open plastic containers (volume 80 ml) that were placed in the at the ends of each row (i.e., at distances of ca 3.5 m between emission sites) at a height of ca 30 cm above the plant surface (**Figure 1**).

##### 2.2.2.2 Fungal challenge

Challenge with *C. lindemuthianum*. Following earlier studies (Quintana-Rodriguez et al., 2015), a suspension of 10^6^ conidia mL^-1^ in distilled water with 0.1% TWEEN was used to challenge the plants by spraying approximately 10 mL of this suspension directly on both surfaces of the leaves. To obtain conidia, mycelia of *C. lindemuthianum* were transferred to new Petri dishes with potato dextrose agar (PDA) medium and maintained at 28°C in the dark. After 2 weeks, conidial suspensions were prepared by washing the mycelium gently in distilled water with 0.1% Tween (Sigma, St. Louis, MO, USA) and diluted 1:10, 1:100 and 1:1000, to adjust and the concentration by counting conidia in aliquots in a Neubauer haematocytometer (Hausser scientific, Horsham, PA, USA).

##### 2.2.2.3 Nonanal followed by fungal challenge

Both treatments were applied as described above, that is, plants were challenged after the 48 hrs of exposure to nonanal.

##### 2.2.2.4 Control

At the same time points as described above, we collocated nonanal-free lanolin paste in the control plots as described above and two days later, plants were mock-challenged by spraying 10 mL distilled water with 0.1% Tween per plant.

We are aware of the fact that mixing plants of different treatments would be statistically desirable, but the volatile nature of nonanal and the potential induction of VOC emission by this treatment, fungal challenge, or both, make a blocked design mandatory.

#### 2.2.2 Treatments of F1 plants (Trial 1).

To study the effects of maternal treatments on anthracnose resistance of the offspring, 25 randomly selected seeds per maternal treatment were sown in each of four spatially separated plots: two for the control group and two dedicated to challenge with *C. lindemuthianum*. The rate of seedling emergence was determined three weeks after sowing, and six weeks after emergence, the plants in two of the plots were challenged with *C. lindemuthianum* as described above.

### 2.3 PR gene expression and VOC emission (Trial 1)

#### 2.3.1 PR gene expression: sampling and sample processing

In the first trial, leaf samples aimed at quantifying the expression of PR-1 and PR-4 were collected from F0 plants at each of four time points: 0 h (before starting the exposure to nonanal), 48 h (after the exposure to nonanal, i.e., directly before challenge with *C. lindemuthianum* where applicable; 72 h (24 h after challenge) and 168 h (five days after challenge). From these plants, we sampled n = 5 individual plants per treatment x time point combination (5 x 4 x 4 = 80 samples in total). Since F1 plants were only challenged with *C. lindemuthianum* but not exposed to nonanal, samples from these plants were collected at three time points: 0 h (before challenge), 24 h (directly after challenge, where applicable) and 144 h (5 days after challenge), sampling n = 3 individual plants per maternal treatment x offspring treatment x time point combination (4 x 2 x 3 x 3 = 72 samples in total). In all cases, leaves were collected from the plants in the field, immediately submerged in liquid nitrogen, ground with mortar and pestle, and stored at -80°C.

To extract RNA, 100 mg of frozen ground tissue was mixed with 900 µL of TRIzol ^®^ reagent (Invitrogen, Carlsbad, CA, USA) for 1 minute. Then, 900 µL of chloroform: isoamyl alcohol 24:1 (Sigma-Aldrich Merck KGaA, Darmstadt, Germany). Following centrifugation at 13,000 g for 10 minutes at 4°C, the supernatant was separated and transferred to another Eppendorf tube, re-suspended in 900 µL of phenol: chloroform: isoamyl alcohol 25:24:1 (Sigma-Aldrich Merck KG, Darmstadt, Germany) and mixed in a vortex (Vortex-Genie 2, Scientific Industries, Bohemia, New York, USA) for 1 min. Following centrifugation at 13,000 g for 10 min at 4°C, the supernatant was separated and transferred to another Eppendorf tube, to repeat the last step one more time. RNA concentration was measured with a NanoDrop (Thermo Scientific, Wilmington, DE, USA), and its integrity and concentration were determined by electrophoresis in agarose gels.

#### 2.3.2 RT PCR and transcript quantification

Reverse transcription was performed using 1 µL of DNase-treated RNA as template, with oligo dT and Super- Script II reverse transcriptase (Invitrogen, Carlsbad, CA, USA). The reaction mixture containing 1 μL of Oligo(dT)12-18 (500 μg mL-1), 1 μL of the RNA sample at 100 ng μL-1, 1 μL of dNTP Mix (10 mM each) and sterile, distilled water to 12 μL was heated to 65°C for 5 min and quickly chilled on ice. After brief centrifugation, we added to each sample 4 μL of 5X First-Strand Buffer, 2 μL of 0.1 M DTT and 1 μL of RNaseOUT™ (Cat. No. 10777-019) (40 units μL-1). The samples were mixed and incubated at 42°C for 2 min. Next, we added 0.5 μL (100 units) of SuperScript™ II RT distilled water to 20 μL final volume and mixed gently by pipetting. The samples were incubated at 42°C for 50 min and reaction was stopped by heating to 70°C for 15 min.

Subsequently, PCR was performed in a Thermal Cycler C1000 Touch™ (BioRad) by incubation of an aliquot of cDNA with specific oligonucleotides. The oligonucleotides used were the following (Quintana-Rodriguez et al., 2015): ACTIN, 5´GGTCGTCCTCGTCACACTGG3´ and 5´GGCATGTGGGAGAGCATAACC 3´; PR-1, 5´AAGACGC CGATACCATCTTCC3´ and 5´CCAGAAGGTATGCCTCTACGG3´ and PR-4, 5´AATGTTGTGGTGAGGGATGGC3´ and 5´CTTTGCATCCTTTGGGCACC3´. The PCR reaction was performed using DNA polymerase (Invitrogen) and the following program: an initial cycle of denaturalization at 95°C for 3 min followed by 29 cycles as follows: 20 cycles at 95°C for 30 s, alignment at 52°C for 45 s, extension at 72°C for 30 s; 9 cycles at 95°C for 30 s, alignment at 55°C for 45 s, extension at 72°C for 1 min and a final extension at 72°C for 10 min. PCR products were separated by gel electrophoresis on 1% agarose gels and photographed. Transcript levels were determined with the IMAGE LAB software (http://www.bio-rad.com/en-ru/product/image-lab-software) and expression levels calculated using actin mRNA as the reference.

### 2.4 Degree of infection (both trials)

Degrees of infection were determined as colony forming units (CFUs) obtained from homogenized leaf tissue. Earlier studies demonstrated that this method and the determination of infection levels by quantifying the fungal membrane lipid, ergosterol, gives very similar results (Quintana-Rodriguez et al., 2015). In the first trial, one randomly selected leaf was collected directly before and seven days after challenging the plants with the fungus *C. lindemuthianum*, from each of 20 plants per treatment (n = 20 biological replicates). In the second trial, one randomly selected leaf per plant was collected seven days after challenge from five randomly selected plants per treatment, due to the much higher numbers of plants in this trial (n = 5 biological replicates). Leaves were weighed (fresh weight) and the sample was homogenized in distilled water. This stock homogenate was diluted 1: 10, 1: 100 and 1: 1,000, and 0.1 mL of the stock homogenate and of each dilution were spread on solid PDA plates, to count emerging colonies four days later. Numbers of CFUs were related to the original fresh mass and the results of the four dilutions per sample were averaged.

In the second trial, we quantified phenotypic disease symptoms in addition to CFUs. Seven days after challenging the plants with the fungus *C. lindemuthianum*, one randomly selected leaf per plant was removed and scanned using a printer equipped with scanning function (Brother DCP-1602). The diseased area was quantified as areas with necrotic lesions on the abaxial surface of the leaf relative to the total area of the leaf, using the Software Image J (https://imagej.nih.gov/ij/).

### 2.5 Seed yield (both trials)

In both trials, we quantified seed yield at the end of the reproductive phase, i.e., when plants started to dry naturally. Therefore, harvesting dates varied between 12 and 13 weeks post germination in the second trial, depending on the variable phenology of the different cultivars. In the first trial, we first counted the number of plants that had survived until reproduction. Seven randomly selected plants per treatment were harvested individually, the pods were removed and opened manually, and the seeds dried in an oven at 50°C for two days, to determine seed dry weight per plant. In the second trial, five plants per treatment and genotype were harvested completely (i.e. including the roots) and pods were removed. Both the plants and the seeds produced per plant were dried individually in an oven at 50°C for two days, to determine total plant dry weight at harvest stage and seed yield.

### 2.6 Phenolic compounds and antioxidants (Trial 1)

#### 2.6.1 Extraction and determination of phenolic compounds

For the photometric determination of the content of phenolic compounds and antioxidants, we first prepared a methanolic extract (Cardador-Martínez et al., 2002). Samples of 200 mg of ground bean seeds were mixed in a 50 mL tube with 10 mL methanol (HPLC, 99.98%) and extracted 24 h in the dark at 25°C on an orbital shaker (Orbit 1000 model S2030-1000; Labnet, Woodbridge, NJ, USA) at 200 rpm. After centrifugation at 5,000g for 10 min at 4°C (Sorvall Biofuge Primo R model 75005448; Thermo Scientifc, Osterode, Germany), aliquots of the supernatant were used for the photometric assays.

The assays followed established protocols: flavonoids were determined following (Oomah et al., 2005), phenols following (Singleton et al., 1999), condensed tannins following (Feregrino-Pérez et al., 2008), and antioxidant activity following (Brand-Williams et al., 1995; Fukumoto and Mazza, 2000; Nenadis et al., 2004). We used reagents from Sigma and microplate readers to quantify absorbance (flavonoids: Multiskan Go model 51119300 from Thermo Scientific, Vantaa, Finland; all other assays: Multiskan Ascent model 51118307, Thermo Electron Corp).

For the determination of flavonoids, 50 µL of the methanolic extract were mixed in a 96-well plate with 180 µL of distilled water and 20 µL of a 1% solution of 2-aminoethyldiphenylborate, and extract absorbance at 404 nm was compared with that of a rutin standard curve (prepared with 0, 0.02, 0.05, 0.1, 0.25 y 0.5 mg mL^-1^ rutin in methanol), to express flavonoid content as miligrams of rutin equivalents per gram of sample dry mass.

For the quantification of total phenolics, 40 µL of the methanolic extract were mixed in a 96-well plate with 250 μL of Folin-Ciocalteu reagent (1 N) and 1,25 mL of a 20% Na_2_CO_3_ solution. After 2h in the dark, extract absorbance at 760 nm was compared with that of a gallic acid standard curve (prepared with 0, 1, 2, 4, 5, 8, 10, 16 y 20 mg mL^-1^ gallic acid in methanol), to express the content of phenols as milligrams of gallic acid equivalents per gram of sample dry mass.

For the quantification of condensed tannins, 50 µL of the methanolic extract were mixed in a 96-well plate with 200 µL of 0.5% vanillin reagent (1% vanillin and 8% HCl 1:1 in methanol), and the absorbance at 492 nm was compared with that of a (+)-catechin standard curve (up tp 0.1 mg ml^-1)^, to express the content of condensed tannins as mg (+)-catechin equivalents per gram of sample dry mass. To correct for potential interference from natural pigments in bean, a blank sample was prepared by subjecting the original extract to the same conditions of reaction without the vanillin reagent.

#### 2.6.2 Antioxidants

For the determination of the overall activity of antioxidants, we used assays based on the scavenging of the 2,2-di (4-tert-octylphenyl)-1-picrylhydrazyl (DPPH) radical to quantify the radical scavenging activity (RSA) and the 2,2′-azinobis (3-ethylbenzothiazoline-6-sulfonate) (ABTS) radical to quantify the Trolox equivalent antioxidant capacity (TEAC, here termed AC). We prepared a solution of DPPH (150 *µ*M) in 80% methanol and added 200 *µ*L of this DPPH solution to 50 μL of sample in a 96-well flat-bottom visible light plate. After 30, 60, 75, 90 and 120 min in the dark at 20°C, absorbance at 532 nm was compared with that of a 6-hydroxy-2,5,7,8-tetramethylchroman-2-carboxylic acid (Trolox) standard curve (range 0.05–0.8 mM). RSA was estimated by quantifying the decrease of the absorption at 532 nm of the DPPH solution after addition of the antioxidant and calculated following Equation 1, where A_s_ is the absorption of the sample and A_c_ is the absorption of the control.


(1) $$RSA=\left(1–A_{s}/A_{c}\right)\text{ x }100$$

The ABTS solution was prepared by reaction of 5 mL of a 7 mM aqueous ABTS solution and 88 *µ*L of a 140 mM (2.45 mM final concentration) potassium persulfate (K2S2O8) solution. After storage in the dark for 12 h at room temperature, the radical cation solution was further diluted in ethanol until the initial absorbance value of 0.7 ± 0.05 atb734 nm was reached. Subsequently, 225 *µ*L of the ABTS solution and 25 *µ*L of sample were mixed in 96-well plates to quantify the absorbance at 734 nm from 0 - 90 min. AC was calculated following Equation 2, where Ai is the initial absorption of the sample and A_f_ is the final absorption.


(2) AC=((Ai−Af)/(Ai)) x 100

### 2.7 Estimating net profit (both trials)

To estimate of the potential economic profit of a yield increase achieved by nonanal, we estimated the costs of this treatment and the economic value of the grain yield per hectare. The basal production costs (tilling, watering and fertilizing) were estimated as 9,900 MXN per ha and season, and the additional costs of the nonanal-treatment were calculated based on the real costs of the material used per release point (2g lanolin, 20mg nonanal, 1 plastic cup, 1 wooden stick) and extrapolating this cost to a hectare, assuming 8,300 release points ha^-1^ (See **Supplementary Text S1** and **Supplementary Table S1** for details).

Similarly, we extrapolated our yield data (in g seed mass plant^-1^) assuming 83,000 plants ha^-1^ and calculated hypothetical minimum and maximum gain based on the governmentally guaranteed price of 21,000 MXN t^-1^, and on prices found in Mercado Libre (https://www.mercadolibre.com.mx). According to sernagrp.com, Mercado Libre is the dominating online platform for ecommerce in México that attracts ca 140 million visits per month (Serna, 2022).

### 2.8 Statistical analyses

Statistical analyses were performed using MINITAB® Statistical Software and Rstudio. Rates of survival and offspring emergence in trial 1 were analysed using unifactorial ANOVA in trial 1 and trial 2, with posthoc Tukey HSD.

## 3 Results

### 3.1 Effects of nonanal on F0 plants (Trial 1)

#### 3.1.1 Nonanal primes PR genes and reduces fungal infection

**Figure 2:**
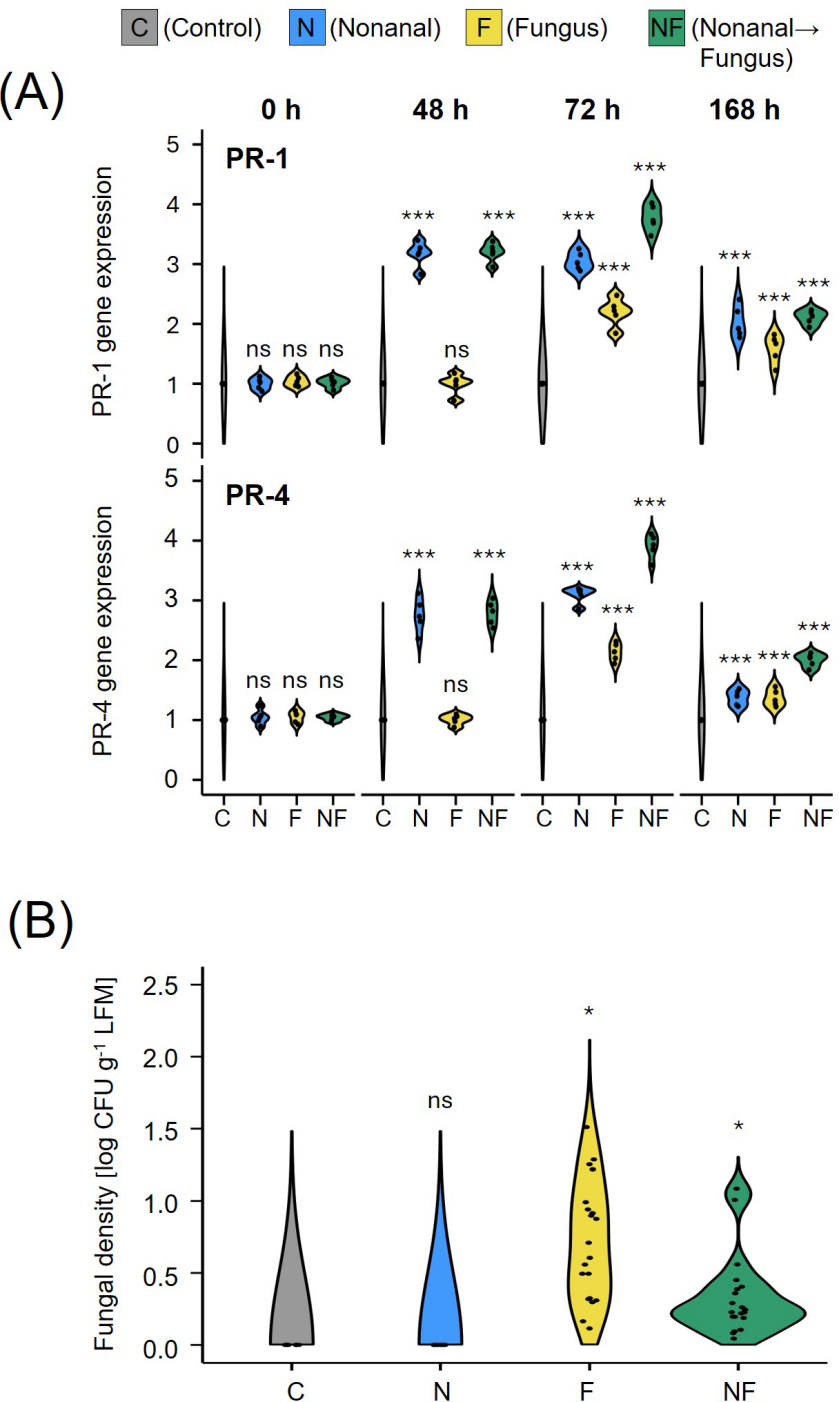
Nonanal exposure primes PR genes and enhances resistance to anthracnose. **(A)** Expression (mRNA) levels of PR-1 and PR-4 in leaves of Flor de Junio Marcela (FJM) bean plants were quantified before exposure to nonanal (0 h), before challenge with *Colletotrichum lindemuthianum* (48 h), 24 h after challenge with *C lindemuthianum* (72 h), and 5 days after the challenge with *C lindemuthianum* (168 h). **(B)** The fungal density of *C lindemuthianum* in leaves of Flor de Junio Marcela bean plants was quantified seven days after challenge (7dac) in colony forming units (CFUs). Violins in A show the density curves of the expression levels of each gene at each time point expressed as a ratio relative to the level found at 0 h in the controls, which was set to 1. Violins in B show the density curves of CFU numbers per gram of leaf fresh mass on a logarithmic scale. Colours of violin plots indicate the treatments: grey = C (control, sprayed with distilled water), blue = N (nonanal, 48 h exposure to nonanal), yellow = F (fungus, challenge with *C. lindemuthianum*); green = NF (nonanal exposure followed by challenge with *C. lindemuthianum*). Asterisks above the violins represent statistically significant differences between a treatment and the control [*p<0.05, ***p< 0.001 according to ANOVA followed by Tukey posthoc tests, ns, not significant, p ≥ 0.05, n = 5 biological replicates in **(A)** and 7 biological replicates in **(B)**].

Semi-quantitative RT-PCR of leaf samples collected from non-challenged plants revealed that exposure to nonanal induced the expression of the two marker genes: after 48 h of exposure, the mRNA levels of PR-1 and PR-4 were more than 3-fold higher than before exposure, and they remained at this level until 72 h (treatment N, see **Figure 2A**). We observed a significant further increase (to ca 3.5-fold over control for PR-1 and ca. 4-fold over control for PR-4) in plants that were subsequently challenged with *C. lindemuthianum* (Treatment NF). Challenge with *C. lindemuthianum* of not nonanal-exposed plants (treatment F) also caused a significant (ca. 2-fold) increase in mRNA levels of PR-1 and PR-4 within 24 h, but this increase was significantly lower than the increase after challenging nonanal-exposed plants (**Figure 2A**).

Quantifying infection rates as numbers of CFUs revealed significant differences among treatments (p*<* 0.05, **Figure 2B**). Plants exposed to nonanal prior to challenge with *C. lindemuthianum* (treatment NF) showed significantly lower infection rates, averaging ca. 50% of the values observed after challenge alone (treatment F). No CFUs of *C. lindemuthianum* could be detected in unchallenged plants (treatments C and N, **Figure 2B**).

#### 3.1.2 Nonanal increases plant survival, seed yield and seed phenolic compounds

Nonanal exposure was associated with significant increases in the survival rate of the plants and in seed yield, independently of whether the plants had been challenged with *C. lindemuthianum*. The number of plants out of the 200 seeds sown per plot that survived until harvest differed significantly among treatments (p< 0.05, chi-square test): on average, 98 plant survived on the control plots (C), 109 on the nonanal-exposed plots (N), 105 on the fungal challenge plot (F), and 147 plants survived on the plot where plants had been exposed to nonanal before challenge (NF).

Nonanal exposure and challenge with *C. lindemuthianum* were also associated with a significant increase (by ca 40% over controls) in the level of phenolic compounds in the seeds (**Table 1**). However, both treatments together had the opposite effect (decrease to less than 50%). Nonanal exposure and fungal challenge also increased the antioxidant and radical scavenging activity, although this effect was significant only for fungal challenge, and all three treatments were associated with strongly decreased contents of flavonoids and slightly decreased contents of condensed tannins (to 40 - 50% and 80 – 90% of control levels, respectively) (**Table 1**).

**Table 1:** Levels of phenolic compounds and antioxidants in FJM beans.

Treatment	Phenolics [mg g^-1^]	Flavonoids [mg g^-1^]	Tannins [mg g^-1^]	AC [%]	RSA [%]
**C**	50.22^b^	21.35^a^	1.82^a^	34.8^b^	56.5^b^
**N**	73.53^a^	14.69^b^	1.70^a^	36.2^b^	60.4^b^
**F**	72.00^a^	10.05^c^	1.61^ab^	56.5^a^	76.7^a^
**NF**	19.46^c^	8.36^d^	1.55^b^	19.0^c^	38.9^c^

Levels of total phenolic compounds, flavonoids and condensed tannins are indicated in equivalents of mg gallic acid, rutin and catechin, respectively, per gram sample dry mass, while the antioxidant activity (AC) and the radical scavenging activity (RSA) are indicated as percent of the inhibition of the ABTS and the DPPH radical. Treatments were C (control, sprayed with distilled water), N (nonanal, 48 h exposure to nonanal), F (fungus, challenge with *C. lindemuthianum*) and NF (nonanal exposure followed by challenge with *C. lindemuthianum*), and different letters indicate significant differences among treatments (p < 0.05 according to ANOVA followed by Tukey post-hoc tests, n = 3 biological replicates).

**Figure 3:**
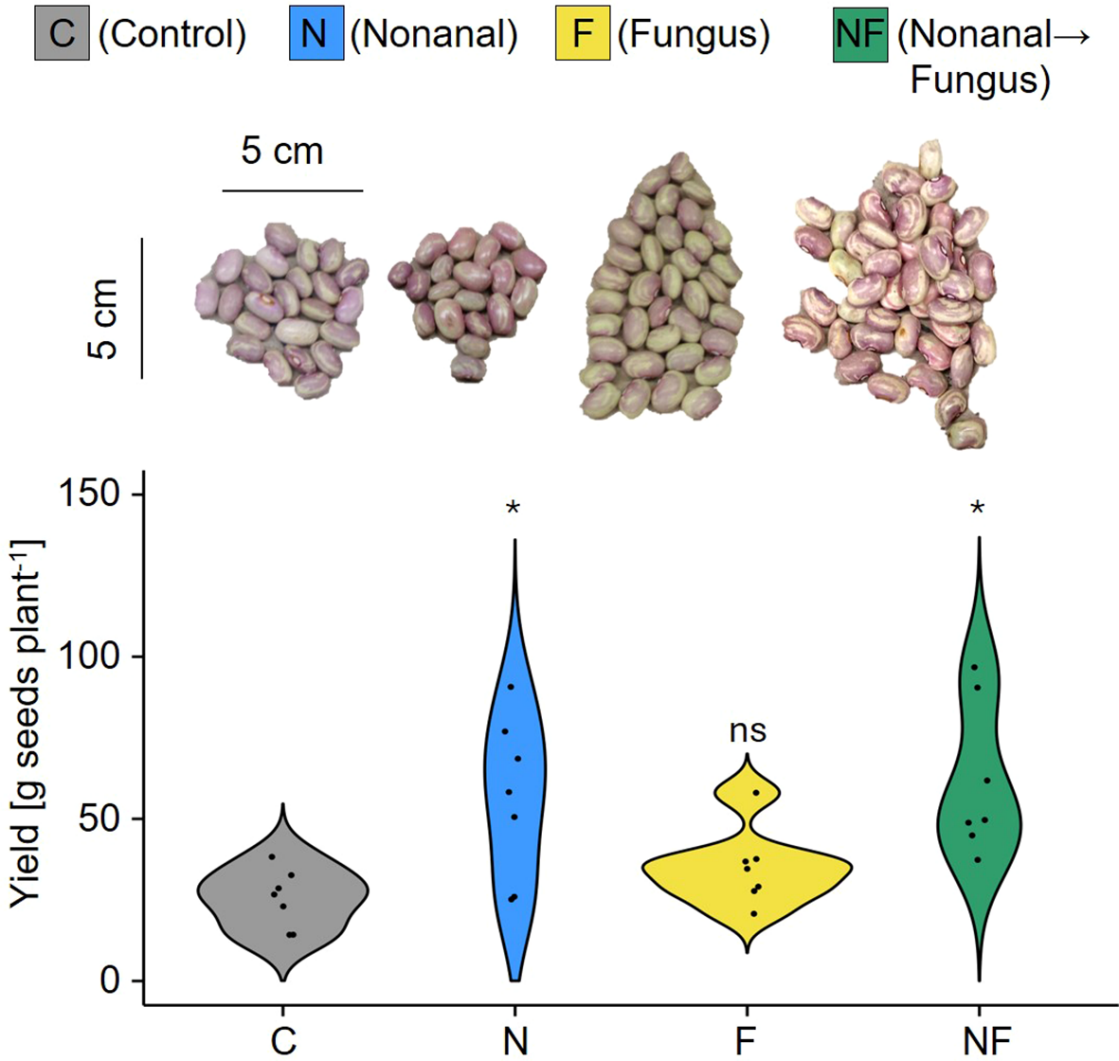
Nonanal enhances seed yield in FJM bean. Seed yield was determined as total dry mass of the seeds produced per each plant and is depicted as density curves. Colours of violin plots indicate the treatments: grey = C (control, sprayed with distilled water), blue = N (nonanal, 48 h exposure to nonanal), yellow = F (fungus, challenge with *Colletotrichum lindemuthianum*); green = NF (nonanal exposure followed by challenge with *C. lindemuthianum*). Asterisks above the violins represent statistically significant differences between a treatment and the control (*p< 0.05 according to ANOVA followed by Tukey posthoc tests, ns = not significant, p ≥ 0.05, n = 7 biological replicates).

Most importantly, the treatments had a significant effect on seed yield (p< 0.05, **Figure 3**). *Post-hoc* tests confirmed significantly higher seed yield for nonanal-exposed plants, both for anthracnose-free plants (from ca. 25 g plant^-1^ in the controls to ca 56 g plant^-1^ in nonanal-exposed plants) and for plants that had been challenged with *C. lindemuthianum* (from ca 34 g plant^-1^ in challenged plants to 61 g plant^-1^ in plants that had been exposed to nonanal before challenge (**Figure 3**).

#### 3.1.3 Nonanal exposure can increase net economic gain obtained with FJM beans

**Figure 4:**
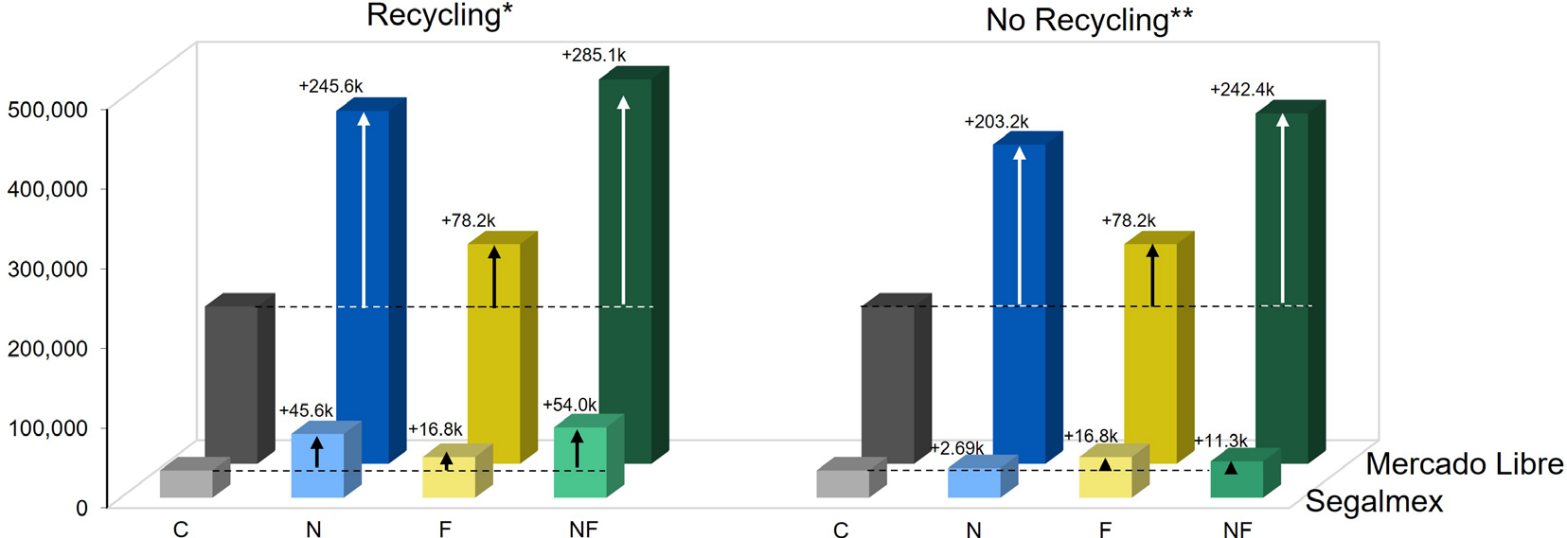
Nonanal treatment can generate a net economic gain. The net economic balance that would have been achieved under the different treatments was estimated based on the different treatment costs and assuming that the product is sold either to the governmental institution, Segalmex, or at an elevated price in Mercado Libre. Columns indicate net gain in Mexican Pesos per hectare [MXN ha^-1^], colours of columns indicate the treatments: grey = C (control, sprayed with distilled water), blue = N (nonanal, 48 h exposure to nonanal), yellow = F (fungus, challenge with *Colletotrichum lindemuthianum*); green = NF (nonanal exposure followed by challenge with *C. lindemuthianum*). Arrows and numbers above columns indicate the difference to the control (which is indicated by a horizontal dotted line). * 'Recycling' refers to the re-use of plastic cups and wooden sticks, ** 'No Recycling' refers to one single use of all components of the release points.

Depending on the treatment, the economic value of the harvested beans would have been ca. 44,400 (C), 99,100 (N), 61,200 (F) and 107,500 (NF) MXN ha^-1^ when assuming the governmentally guaranteed price of 21,000 MXN t^-1^ (**Supplementary Table S2**). We estimated the cost of the nonanal-treatment as 7.34 Mexican Pesos (MXN) per release point, which translates to 61,700 MXN ha^-1^, meaning that the net economic gain of the yield increase that was achieved with the nonanal-treatment would have been negligible (**Figure 4**, right panel).

Evidently, a very different balance would result if the beans could be sold at a net price of 98 MXN kg^-1^ in Mercado Libre. At this price, the economic value of the harvested beans would have been 210,0000 (C), 462,000 (N), 285,000 (F) and 501,100 (NF) MXN ha^-1^, generating a significant economic net gain in spite of the treatment costs (**Figure 4**).

Alternatively, to selling at an elevated price, the treatment cost drops to ca. 19,000 MXN ha^-1^ when the plastic cups and the wooden sticks are re-used 10 and 20 times, respectively (**Supplementary Text S1**, **Supplementary Table S1**, **Supplementary Sheet S1**). Assuming the resulting, lower treatment costs, using the nonanal treatment would have generated an economic net gain even when beans are sold to Segalmex (**Figure 4**, left panel).

### 3.2 Transgenerational effects of nonanal (Trial 1, F1 plants)

#### 3.2.1 Seedling emergence

**Figure 5:**
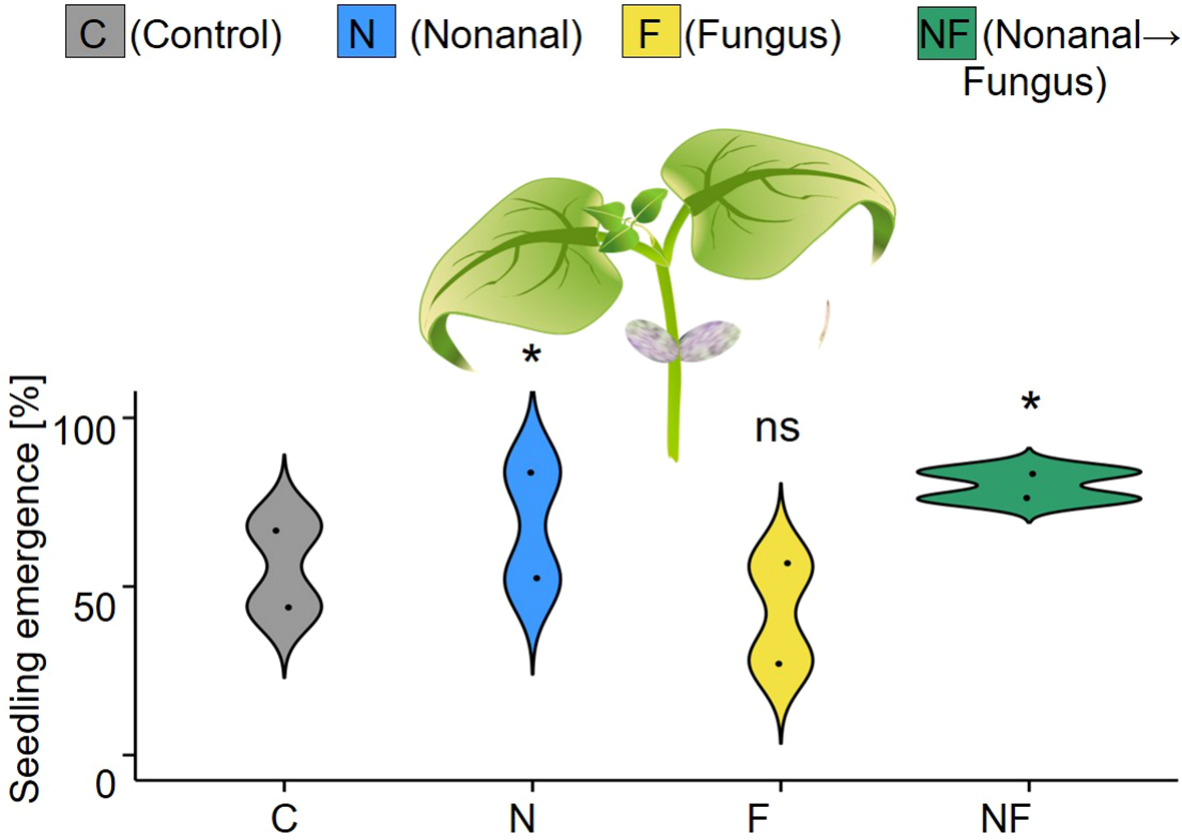
Maternal effects on seedling emergence. Seedling emergence was determined three weeks after sowing as the proportion of seedlings [%] that emerged from the 25 seeds per maternal treatment sown on each of two independent plots per treatment. Colours of violin plots indicate the treatments: grey = C (control, sprayed with distilled water), blue = N (nonanal, 48 h exposure to nonanal), yellow = F (fungus, challenge with *Colletotrichum lindemuthianum*); green = NF (nonanal exposure followed by challenge with *C. lindemuthianum*). Asterisks above the violins indicate statistically significant differences between a treatment and the control (* p< 0.05 according to chi-square test, n = 2 biologically independent replicates).

Emergence was the first factor for which a significant maternal treatment effect became detectable. At three weeks after sowing, plantlets had emerged from 58% (C), 70% (N), 42% (F) or 80% (NF) of the 2 x 25 seeds per maternal treatment that had been sown in two plots (see **Figure 5**).

#### 3.2.2 Maternal nonanal exposure triggers transgenerational priming of PR genes and enhances anthracnose resistance in offspring plants

**Figure 6:**
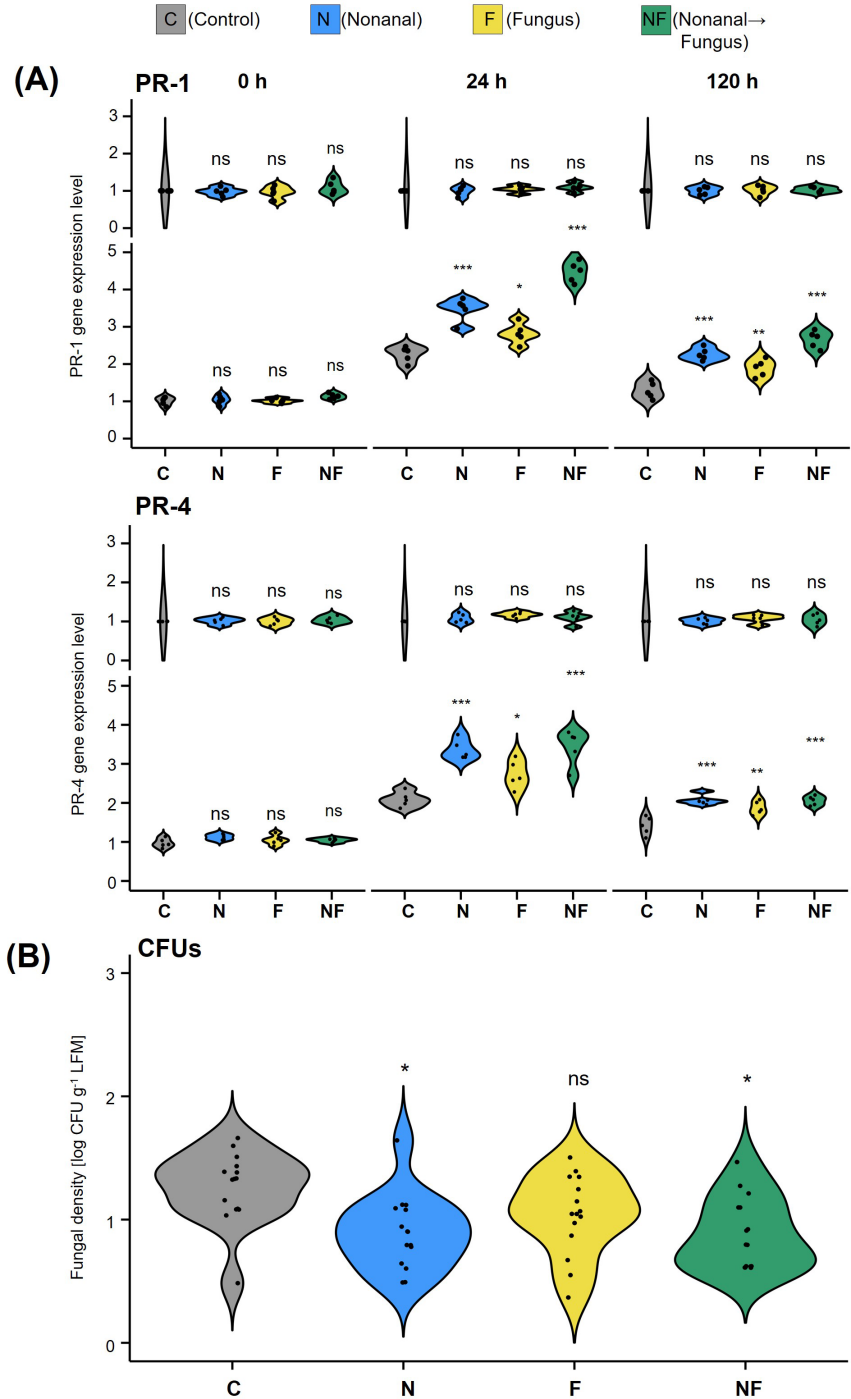
Maternal nonanal exposure primes PR gene expression for an enhanced resistance to anthracnose in the offspring **(A)** Expression of PR1 and PR4 and **(B)** Fungal density of *Colletotrichum lindemuthianum* in the offspring of Flor de Junio Marcela (FJM) bean plants directly before (0 h) and 24 h and 5 days (144 h) after challenge with *C*. *lindemuthianum* in A, and 7 days (168 h) after challenge with *C. lindemuthianum* in **(B)**. Violins in **(A)** show the density curves of the expression levels of each gene at each time point expressed as a ratio relative to the level found at 0 h in the controls, which was set to 1. Violins in **(B)** show the density curves of CFU numbers per gram of leaf fresh mass on a logarithmic scale. Colours of violin plots indicate the maternal treatments: grey = C (control, sprayed with distilled water), blue = N (nonanal, 48 h exposure to nonanal), yellow = F (fungus, challenge with *C*. *lindemuthianum*); green = NF (nonanal exposure followed by challenge with *C*. *lindemuthianum*). Asterisks above the violins represent statistically significant differences between a treatment and the control [*p< 0.05; **p< 0.01; ***p< 0.001 according to ANOVA followed by Tukey posthoc tests, ns = not significant, p ≥ 0.05, n = 3 biological replicates in **(A)** and 5 biological replicates in **(B)**].

The expression patterns of PR1 and PR4 in the F1 generation were similar to those in the F0 generation (**Figure 6A**). In all unchallenged offspring plantlets (C-C, N-C, F-C and NF-C, the first letter indicates the maternal treatment, the second letter the offspring treatment), the expression of both genes was at basal levels, with no detectable differences among plantlets that stemmed from maternal plants subjected to different treatments. At 24 h after challenging with *C. lindemuthianum*, the expression of PR1 and PR4 increased significantly in the offspring of parental plants from all treatments, including the maternal controls. However, while mRNA levels increased ca 2-fold within 24 h in C-F, plants, the increases in N-F and NF-F plants were much stronger (increase by over 4-fold in PR1 and ca. 3.5-fold in PR4), and significantly different from the increases in the other six conditions (p< 0.05 according to ANOVA followed by Fisher LSD posthoc test, n = 5 biological replicates).

Maternal nonanal exposure also had a significant effect (p< 0.05 according to ANOVA) on the infection in challenged offspring plants: The infection rates in the challenged offspring of control plants and of only challenged plants (treatment combinations C-F and F-F) were significantly higher than in the offspring of nonanal-exposed plants (p< 0.05 for treatment combinations N-F and NF-F according to Fisher LSD posthoc test, see **Figure 6B**).

#### 3.2.3 Maternal nonanal exposure enhances seed yield under pathogen pressure.

**Figure 7:**
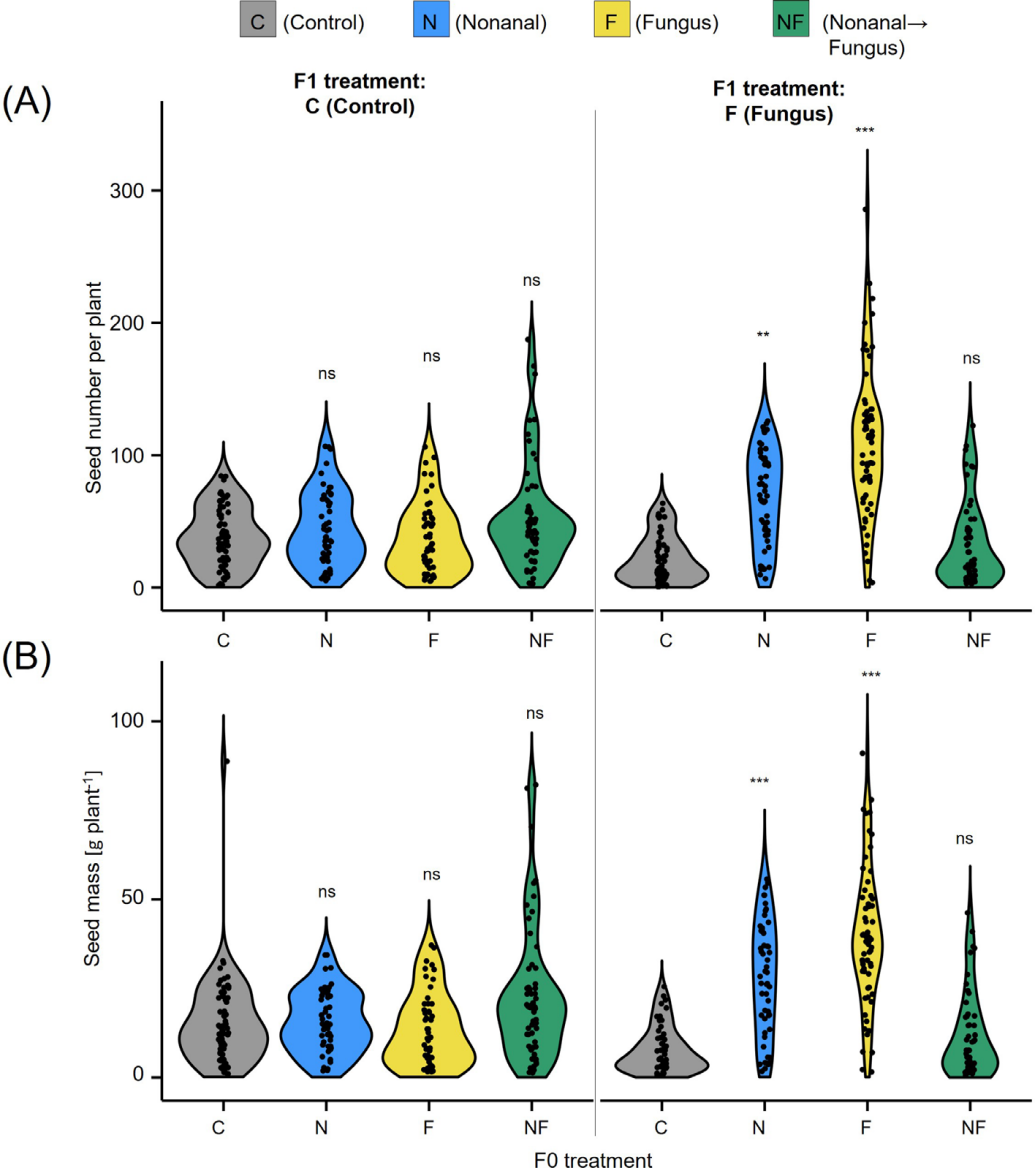
Maternal effects on seed production. **(A)** Seed number and **(B)** seed yield (in gram dry mass) per plant are depicted for the offspring of maternal plants from all four treatments, separately for F1 plants sprayed with distilled water (C, Control) or challenged with *Colletotrichum lindemuthianum* (F, Fungus). Colours of violin plots indicate the maternal treatments: grey = C (control, sprayed with distilled water), blue = N (nonanal, 48 h exposure to nonanal), yellow = F (fungus, challenge with *Colletotrichum lindemuthianum*); green = NF (nonanal exposure followed by challenge with *C. lindemuthianum*). Asterisks above the violins represent statistically significant differences between a treatment and the control (**p< 0.01; ***p< 0.001 according to ANOVA followed by Tukey posthoc tests, ns = not significant, p ≥ 0.05, n = 20 biological replicates).

Overall, the maternal treatment had a significant effect on the seed production in the offspring generation, both when determined as seed number and as seed dry mass (in grams per plant, see **Figure 7**). However, in a more detailed examination we could not detect any significant differences among non-challenged offspring plants, which produced on average ca. 10 – 20 g seed plant^-1^ independently of the treatment to which the maternal plants had been subjected. By contrast, offspring of nonanal-exposed that had been challenged with *C. lindemuthianum* produced ca. 30 g plant^-1^, while the challenged offspring of control plants and NF-plants produced less than 10 g plant^-1^) (**Figure 7**).

### 3.3 Effects of nonanal across cultivars (Trial 2)

#### 3.3.1 Nonanal-induced resistance to anthracnose is reproducible across bean cultivars

**Figure 8:**
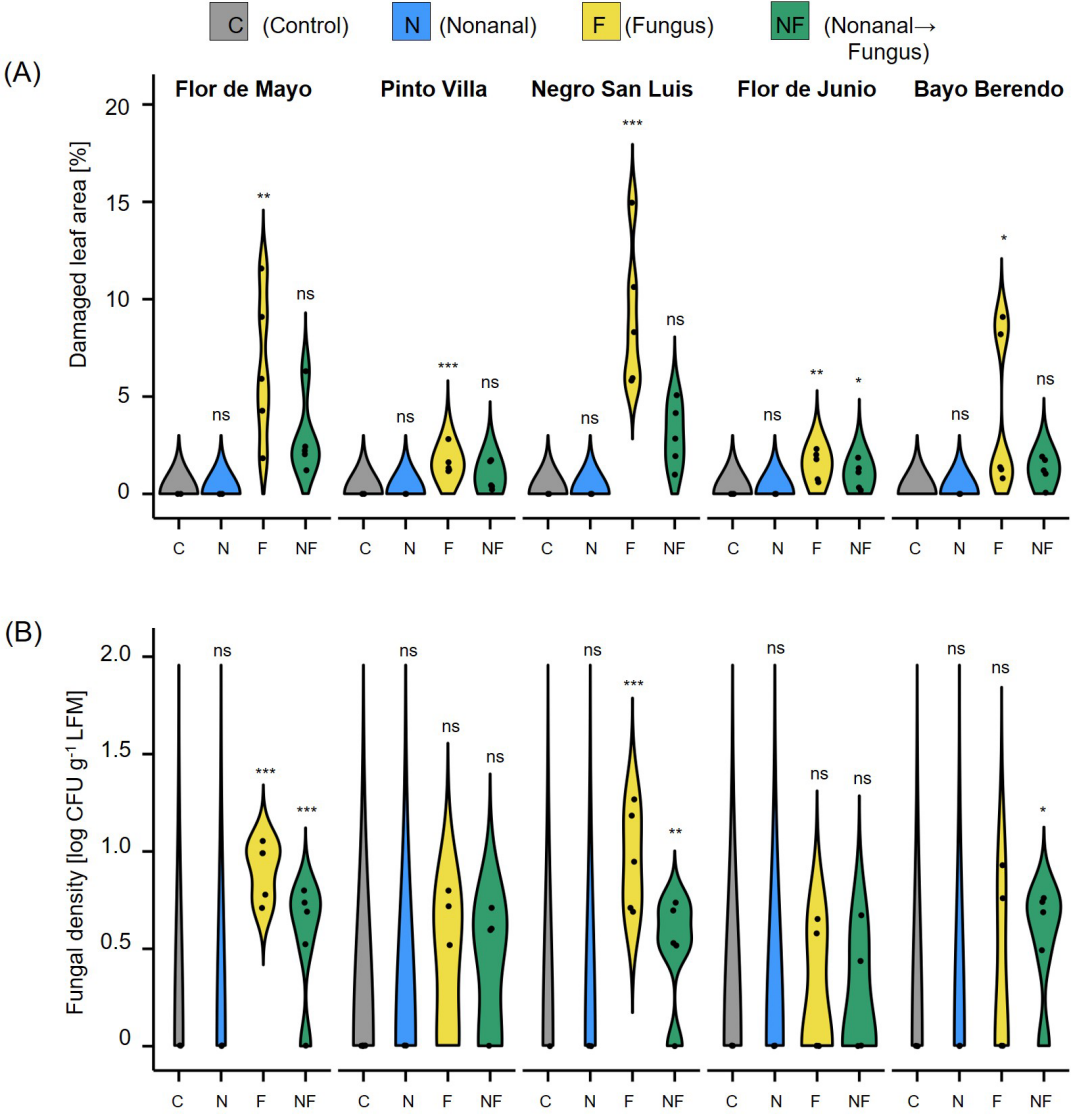
Effects of nonanal on resistance to fungal anthracnose are detectable in different cultivars. **(A)** Phenotypic disease symptoms and **(B)** fungal density in leaves collected 7 days after challenging the plants with *Colletotrichum lindemuthianum*. Cultivars used are Flor de Mayo Anita (FMA), Pinto Villa (PV), Negro San Luis (NSL), Flor de Junio Marcela (FJM) and Bayo Berrendo (BaB). Violins show disease levels quantified as proportion of necrotic areas [in % of the total area] and numbers of colony forming units CFU) per gram of leaf fresh mass on a logarithmic scale (CFUs), respectively. Colours of violin plots indicate the treatments: grey = C (control, sprayed with distilled water), blue = N (nonanal, 48 h exposure to nonanal), yellow = F (fungus, challenge with *C*. *lindemuthianum*); green = NF (nonanal exposure followed by challenge with *C*. *lindemuthianum*). Asterisks above the violins represent statistically significant differences between a treatment and the control (*p< 0.05; **p< 0.01; ***p< 0.001 according to ANOVA followed by Tukey posthoc tests, ns = not significant, p ≥ 0.05, n = 5 biological replicates.

The visual quantification of disease symptoms in *C. lindemuthianum*-challenged plants revealed significant effects of nonanal in four cultivars (*P<* 0.05, according to t test, n = 5 biological replicates), whereas in FJM we observed only a - statistically non-significant - tendency towards reduced leaf damage after nonanal exposure (**Figure 8A**). Similarly, nonanal-exposed plants of all five cultivars showed lower infection rates than only challenged plants, but the differences in the numbers of CFUs reached the level of statistical significance only in three cultivars (FMA, NSL and BB, **Figure 8B**).

#### 3.3.2 Nonanal enhances plant growth and seed yield in different bean cultivars

**Figure 9:**
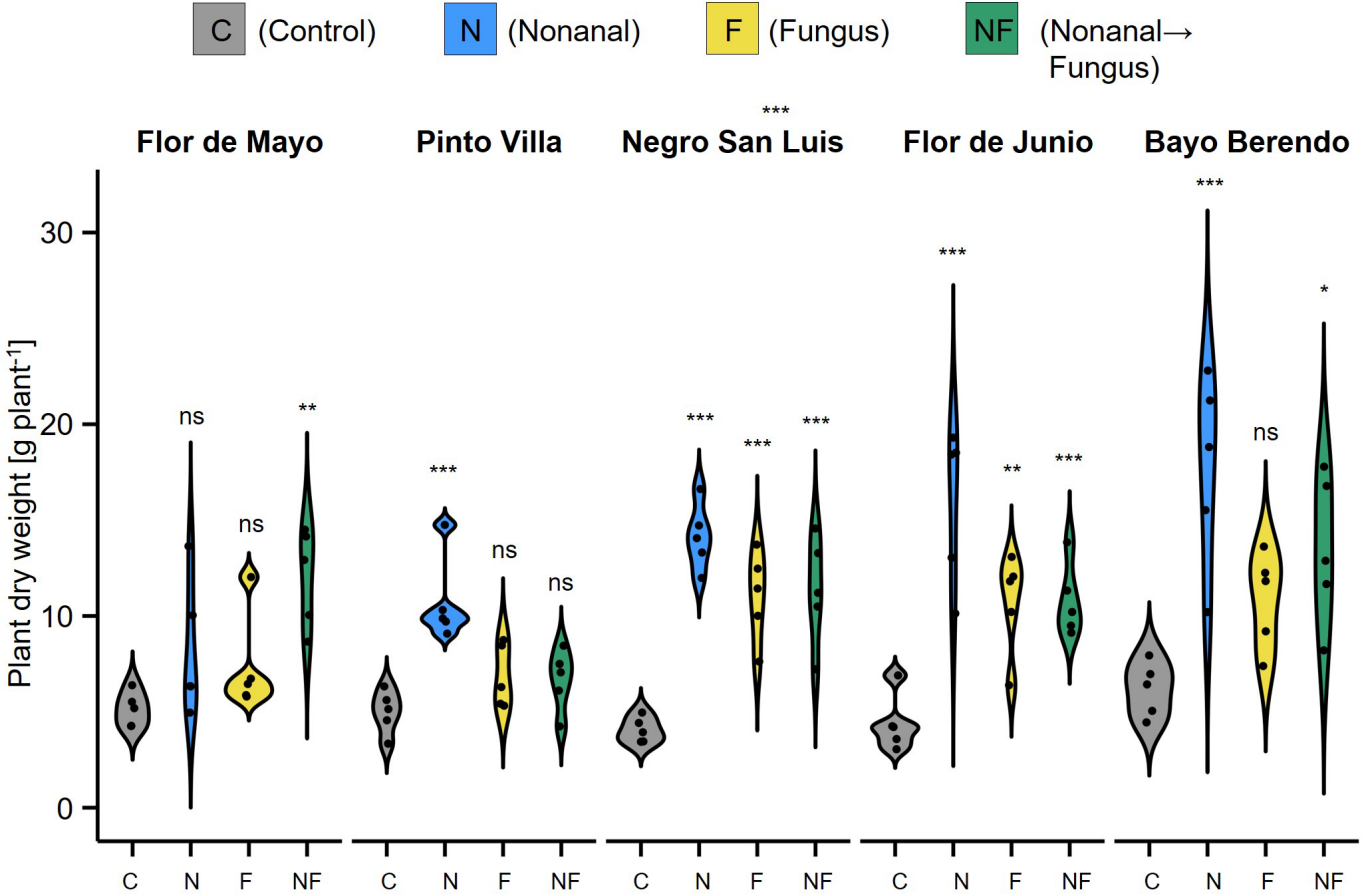
Effects of nonanal on plant size at harvest. The vegetative dry mass at harvest stage was determined as total dry mass of the aerial parts and the roots (but with pots removed) and is depicted in gram per plant as density curves, separately for each cultivar and treatment. Cultivars used are Flor de Mayo Anita (FMA), Pinto Villa (PV), Negro San Luis (NSL), Flor de Junio Marcela (FJM) and Bayo Berrendo (BaB). Colours of violin plots indicate the treatments: grey = C (control, sprayed with distilled water), blue = N (nonanal, 48 h exposure to nonanal), yellow = F (fungus, challenge with *Colletotrichum lindemuthianum*); green = NF (nonanal exposure followed by challenge with *C. lindemuthianum*). Asterisks above the violins represent statistically significant differences between a treatment and the control (*p< 0.05; **p< 0.01; ***p< 0.001 according to ANOVA followed by Tukey posthoc tests, ns = not significant, p ≥ 0.05, n = 5 biological replicates).

In this trial, plants were harvested completely and dried. Therefore, we could test for treatment effects on the total dry weight of bean plants (aerial parts and roots) at the end of the growth cycle, finding that - except for FMA - nonanal-exposed plants had a significantly higher dry weight at harvest than plants subjected to any of the other treatments (**Figure 9**).

**Figure 10:**
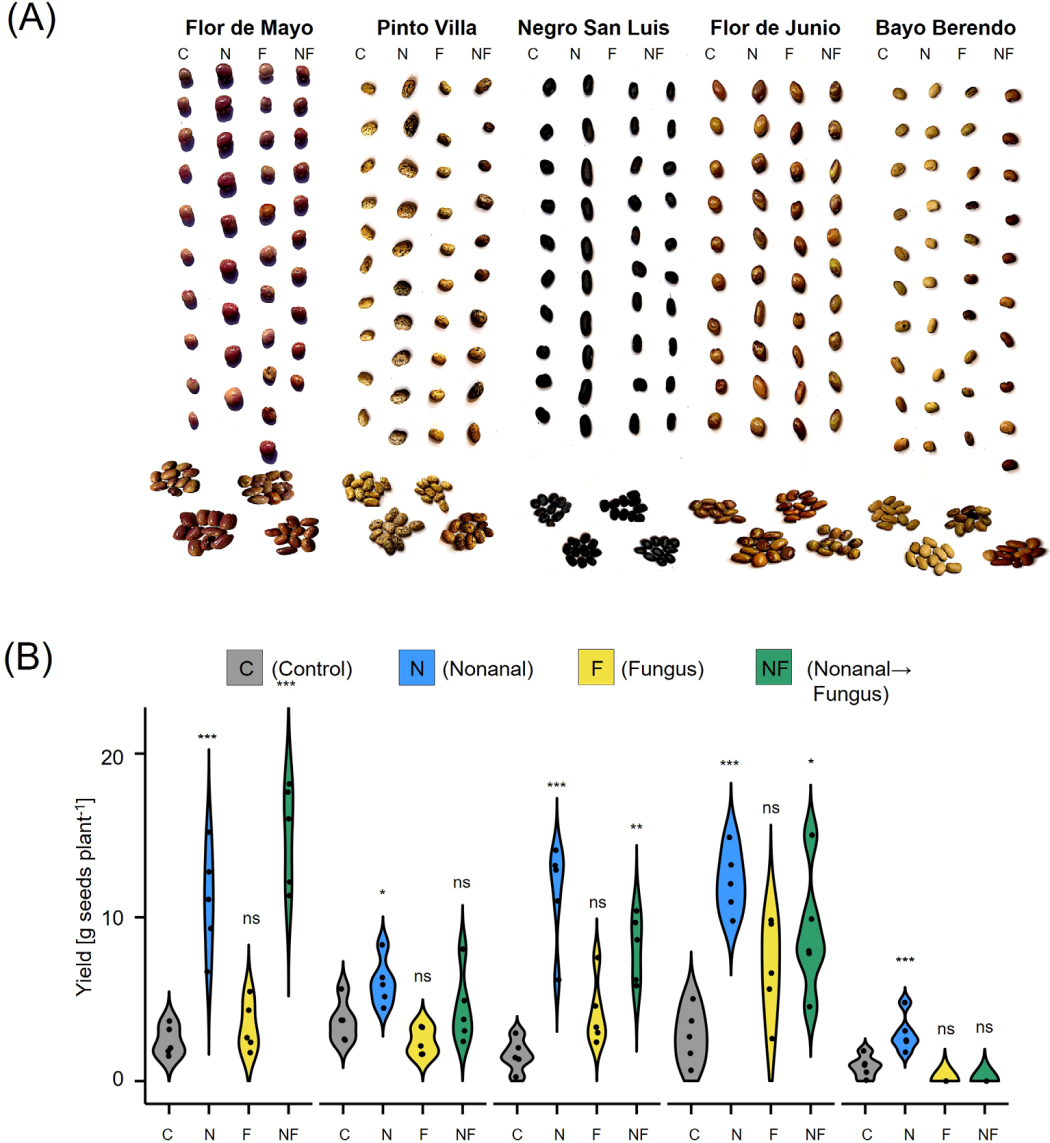
Nonanal increases seed yield and enhances visual quality of beans from different cultivars. **(A)** Photos of ten randomly selected seeds per treatment and cultivar are shown to illustrate the visual differences among seeds from Flor de Mayo Anita (FMA), Pinto Villa (PV), Negro San Luis (NSL), Flor de Junio Marcela (FJM) and Bayo Berrendo (BaB). **(B)** Yield is depicted in gram seeds (dry mass) per plant as density curves, separately for each cultivar and treatment. Colours of violins indicate treatments. Control (C) sprayed with distilled water; challenge with *C. lindemuthianum (*F*)*; exposed to nonanal for 48 h (N) and exposed to nonanal and after challenge with *C*. *lindemuthianum* (NF) in bean genotypes “Flor de Mayo Anita” (FMA),”Pinto Villa” (PV),”Negro San Luis” (NSL), “Flor de Junio Marcela” (FJM) and “Bayo Berrendo” (BaB). Asterisks above the violins represent statistically significant differences between a treatment and the control (*p< 0.05; **p< 0.01; ***p< 0.001 according to ANOVA followed by Tukey posthoc tests, ns = not significant, p ≥ 0.05, n = 5 biological replicates).

Similar tendencies were observed for seed yield (**Figure 10**). Intriguingly, we observed visual differences among the seeds produced by plants from different treatments: seeds from nonanal-exposed plants were larger and exhibited a more homogenous coloration of the surface (**Figure 10A**). Both treatment and genotype had significant effect on total seed yield per plant, with nonanal-exposed (N) plants reaching the highest overall seed yield in four of the cultivars, the exception being FMA (**Figure 10B**). Moreover, challenge with *C. lindemuthianum* after nonanal exposure was associated with significantly higher seed yield in FMA and NSL plants (**Figure 10B**).

#### 3.3.3 Negative economic balance of nonanal exposure for various bean cultivars under low-yield conditions

**Figure 11:**
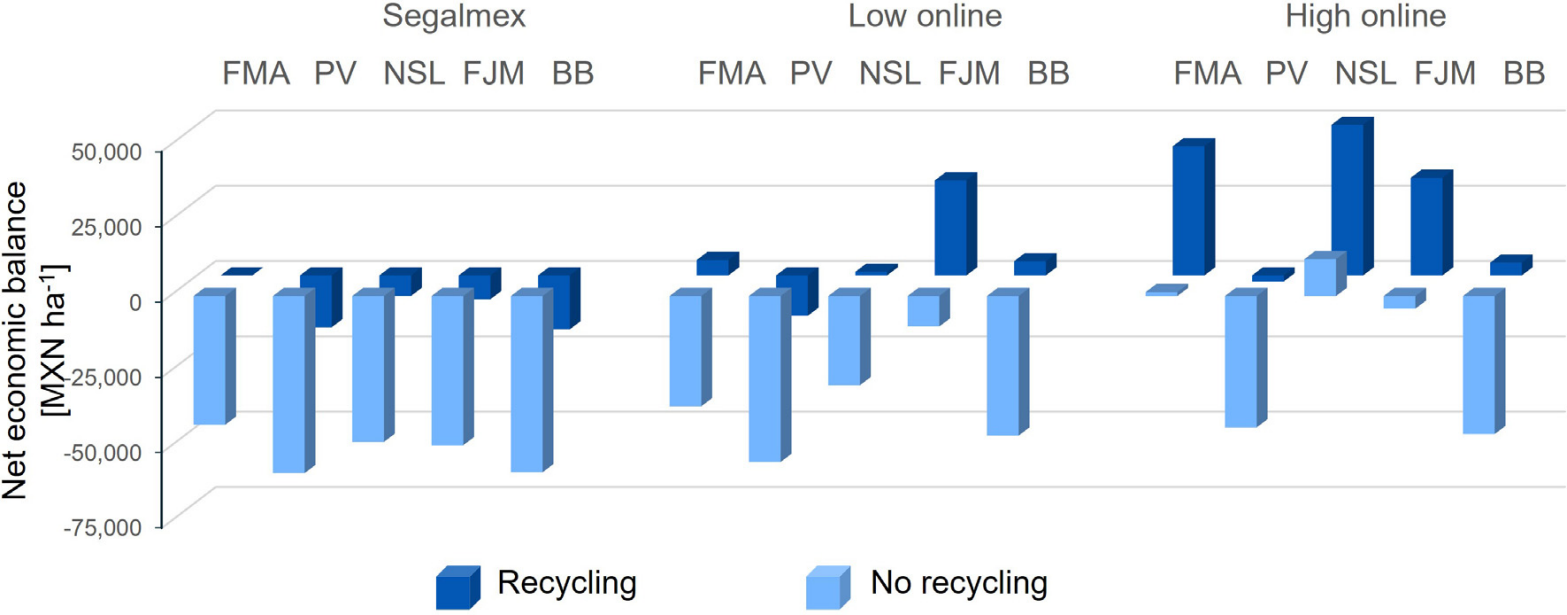
Net economic balance of nonanal treatment for different bean cultivars grown under non-optimum conditions. The net economic balance relative to the control that would have been achieved treating five different bean cultivars under non-optimum conditions (autumn, dry season) with nonanal was estimated based on the treatment costs and assuming that the product is sold either to the governmental institution, Segalmex, or at a moderate or a high price in Mercado Libre. Columns indicate the difference in the net gain as compared to controls in Mexican Pesos per hectare [MXN ha^-1^], bean cultivars used were “Flor de Mayo Anita” (FMA),”Pinto Villa” (PV),”Negro San Luis” (NSL), “Flor de Junio Marcela” (FJM) and “Bayo Berrendo” (BaB), and ‘recycling’ refers to re-using wooden sticks and plastic cups to lower the treatment costs.

Due to the lower absolute yield that was achieved in trial 2, the economic net balance of the nonanal treatment would have been negative for all cultivars if beans were sold to Segalmex (**Figure 11**, left panel, and **Supplementary Table S3**). Even when selling online at a ‘moderate’ price, a net gain would have been achieved only for three of the five cultivars. and only when considering the lower treatment costs resulting when the material is recycled (**Figure 11**, centre panel). Assuming, finally, the higher prices of beans sold as ‘premium quality’, ‘organic’ or ‘agroecological’ combined with lowering the treatment costs by re-using material, nonanal exposure of would have generated a net economic profit for four of the cultivars (**Figure 11**, right panel, and **Supplementary Table S3**).

## 4 Discussion

In the present work, we explored the potential of the volatile aldehyde, nonanal, for an application as a biostimulant that exerts resistance-inducing effects and thereby increases the yield of common bean (Yi et al., 2009; Quintana-Rodriguez et al., 2015). Many attempts to apply VOCs as biocontrol agents have failed because effects observed under controlled laboratory conditions were not reproducible under the more variable field conditions. Since we aimed to perform a translational study, we tested five different cultivars of bean and considered grain yield as the most important response variable. Exposure to nonanal was associated with significantly enhanced grain yield in all five bean cultivars, with yield increases over control ranging from 50% in Pinto Villa (PV) and ca 200% in Flor de Junio Marcela (FJM), Flor de Mayo Anita (FMA) and Bayo Berendo (BB), to an additional 500% more grain yield (gram seed dry mass per plant) in Negro San Luis (NSL) (**Figures 3**, **10**). In FJM, the positive effect on yield could be reproduced in two different seasons. Since we grew plants from seed directly sown into the soil and cultivated these plants with manual watering and hand weeding until harvest, without using any pesticides, we consider our experimental conditions as realistic for Mexican smallholder farmers who cultivate bean for autoconsumption: the purpose of more than 70% of the bean production in the rural environment of México (SAGARPA, 2017; Borja Bravo et al., 2021). Evidently, these conditions are subject to strong variations in biotic and abiotic factors, which could not even be monitored, and less so, controlled. The consequence are variable results and low test power and in the end, relatively few statistically significant differences. However, limitations in the available space and (wo)manpower made larger experiments impossible, and smallholder farmers operate under the same limitations. That is, at the very least, we can be confident that all observed effects on the factor of interest (grain yield) occurred under ´real-world conditions’, which strongly enhances the probability that our results can be replicated by the audience of interest: the smallholder farmers. In this sense, our ‘from-the-field-to-the-lab’ approach follows suggestions for the medical field – an aera traditionally hampered by a huge translational gap – for which the influential medical researcher, statistician and metascientist John Ioannidis recommended to increase the heterogeneity in the circumstances of testing (i.e., the opposite of the usual, highly controlled and standardized conditions) as a measure to enhance the generalizability of the study results (Ioannidis et al., 2014).

We tried to self-evaluate our handling of the crop and therefore, we compared our yield values to published data. According to (Beebe et al., 2013), “National averages in Latin America range from 600 to 950 kg ha^-1^”. In trial 1, the F0 generation was cultivated during the rainy season (March – August) 2017 with additional irrigation. Control plants of FJM produced on average 25 g seeds plant^-1^, while nonanal-exposed plants produced 57 g plant^-1^. If extrapolating from our values to a hectare, our yield values were in the upper range of the values obtained in ‘real agriculture’ (**Table 2**). The beneficial effects of nonanal on yield could be reproduced – at very different absolute values – in trial 2, which was performed from August to December 2019, i.e., in the dry season. In bean, yield reductions due to drought may range from 10 to 90%, although most studies report reductions by 50% to 75% of the yield under irrigation (Castellanos-Ramos et al., 2003; Acosta-Díaz et al., 2009; Smith et al., 2019; Godoy Androcioli et al., 2020; Papathanasiou et al., 2022; Mutari et al., 2023) (see **Table 2**). Correspondingly, the yield of nonanal-exposed FJM plants in trial 2 was 10.0 g plant^-1^ versus 2.8 g plant^-1^ in the controls, challenged plants yielded 5.6 g, while nonanal exposure prior to challenge allowed for a yield of 9.8 g seeds plant^-1^. Similarly, the yield we obtained for the other four cultivars tested were lower than reported in the literature for optimum conditions. However, when extrapolating to a hectare, the yield of FJM plants was within the range reported for rainfed conditions and in case of nonanal-exposed NSL, it was even higher (**Table 2**). Thus, we consider our yield as an indication that our growing conditions were sufficiently close to ‘real-world’ conditions to allow for meaningful conclusions.

**Table 2:** Yield of five bean cultivars used in the present study in comparison to published yield values.

	Cultivar	This work				Rainfed	Irrigation	Reference
		C	N	F	NF			
Trial1 1 F0	FJM rainy season kg ha^-1^*	2,900 (25.39)	4,700 (34.96)	5,100 (56.61)	8,100 (61.41)	680-900	1,690-2,900	(Castellanos-Ramos et al., 2003)
Trial 2 (dry season)	FMA kg ha^-1^	200 (2.44)	630 (7.65)	270 (3.31)	900 (10.89)	940	1,380	(Acosta-Díaz et al., 2009)
	PV kg ha^-1^	300 (3.62)	460 (5.49)	260 (3.16)	550 (6.62)	1390	1,520	(Acosta-Díaz et al., 2009)
	NSL kg ha^-1^	130 (1.60)	790 (9.53)	420 (5.09)	740 (8.95)	550	2,250	(Acosta-Díaz et al., 2009)
	FJM kg ha^-1^	230 (2.75)	830 (10.02)	460 (5.59)	810 (9.80)	680-900	1,690-2,900	(Castellanos-Ramos et al., 2003)
	BB kg ha^-1^	75 (0.90)	240 (2.91)	0 (0.00)	0 (0.00)	1,300	2,300	(Acosta-Díaz et al., 2009)
	**Average** (without BB)	**750**	**1,480**	**1,300**	**2,050**	**450**	**1,840**	(Acosta-Díaz et al., 2009)

*For the sake of easier comparisons, the yield values obtained in the present study were extrapolated to kg ha^-1^, the original values (in g plant^-1^) are indicated in parentheses.

### 4.1 Resistance induction or growth promotion?

Nonanal exposure prior to challenging the plants with *Colletotrichum lindemuthianum* significantly reduced leaf damage and fungal infection in challenged plants of three cultivars (**Figure 8**). Resistance to fungal pathogens after pre-exposure to a VOC (or a blend of VOCs) has been reported, e.g., for Arabidopsis (Cordovez et al., 2017), barley (Brambilla et al., 2022; Laupheimer et al., 2023), bean (Quintana-Rodriguez et al., 2015), corn (D'Alessandro et al., 2014), grapevine (Lazazzara et al., 2018; Avesani et al., 2023) and strawberry (Xu et al., 2019). In our study, the decrease in infection rate in FJM was associated with a 2-fold yield increase (**Figure 3**), an observation that could indicate a causal relation between these two effects. However, from a closer analysis of our data we conclude that disease resistance alone is not likely to fully explain the effects on seed yield.

First, in most cultivars, the reduction in infection rates was up to an order of magnitude lower than the increase in yield. Evidently, these two factors cannot be expected to correlate linearly, but still, it seems difficult to envision that a reduction of fungal density by 37% in FMA plants or by 60% in NSL plants (**Figure 6**) is sufficient to explain a 5-fold (FMA) or even 6-fold (NSL) higher seed yield. Second, nonanal exposure increased the plant dry mass at harvest (**Figure 9**) and the yield (**Figures 3**, **10**) even in unchallenged plants. In fact, in four of the cultivars, N-treated plants produced more seeds than the plants from the other three treatments, an observation that clearly indicates the relevance of other factors than the reduction of anthracnose. One of these factors could be resistance to other plant enemies. Nonanal induces the resistance to various bacterial and fungal pathogens (Yi et al., 2009; Quintana-Rodriguez et al., 2015; Brambilla et al., 2022), and it has direct antimicrobial effects (Andersen et al., 1994; Bisignano et al., 2001; Dilantha-Fernando et al., 2005; Rajer et al., 2017; Zhang et al., 2017; Quintana Rodríguez et al., 2018b; Li et al., 2021). Moreover, several studies reported that nonanal attracts parasitoids (Dweck et al., 2010; Yu et al., 2010; Li et al., 2022). Since all experiments were performed in the open field, the induction of resistance to pathogens other than *C. lindemuthianum* and/or the attraction of natural enemies of herbivores have likely contributed to the yield-enhancing effects of nonanal. However, a quantification of visible disease symptoms in trial 2 gave similar results as the quantification of CFUs and therefore, an enhanced resistance to other, non-defined biological enemies was likely not the major reason behind the effects of nonanal on bean yield.

### 4.2 Growth stimulation by a volatile DAMP

The increased plant dry weight at harvest makes it tempting to speculate about a growth-promotion effect. Plant growth promotion is usually reported for microbial VOCs (Schulz-Bohm et al., 2017; Sharifi and Ryu, 2018), but not for plant VOCs. However, care must be taken in this context, because nonanal cannot be strictly characterized as a ‘plant-VOC’. In fact, nonanal has been identified as a fungal volatile that promotes the growth of lettuce (*Lactuca sativa*) (Asghar and Kataoka, 2023) and as a bacterial volatile with antifungal activity (Dilantha-Fernando et al., 2005). Non-plant biological sources of nonanal range - besides bacteria and fungi (http://bioinformatics.charite.de/mvoc) - from chicken egg shells to human breath and skin (Jha, 2017; Xiang et al., 2019), and nonanal concentrations increase in skin odour after ozone exposure or burning and in the breath of smokers or patients with lung cancer (Fuchs et al., 2010; Jareno-Esteban et al., 2013; Callol-Sanchez et al., 2017; DeHaan et al., 2017; Jha, 2017; Floss et al., 2022). DAMPs are released from infected, burned or otherwise damaged tissues, that is, in situations that usually require wound closing and tissue regeneration in addition to an immune response. Thus, if nonanal was a textbook example of a volatile DAMP that indicates oxidative damage, its ‘raison d’être’ would call for pro-regenerative properties that could translate to plant growth promotion (Quintana-Rodriguez et al., 2018a). Intriguingly, nonanal also promotes the proliferation of human hair follicular cells via the induction of enhanced levels of the second messenger, cyclic adenosine monophosphate (cAMP) (Park et al., 2020). Evidently, further work will be required to confirm the growth-promoting effect of nonanal and to identify the underlying mechanisms. More importantly, the net positive effects of nonanal on growth and reproduction demonstrate that metabolic or ecological costs of nonanal or the nonanal-induced immunity (Heil, 2001; Heil and Baldwin, 2002) were of minor relevance, at least in our study system.

### 4.3 Transgenerational effects of nonanal might allow production of vaccinated seeds

Although fungal challenge alone increased the expression of PR genes, we observed stronger increases in plants that had been exposed to nonanal before challenge (**Figure 2**). This pattern is consistent with a priming by nonanal (Martínez-Medina et al., 2016). Intriguingly, defence priming in plants is frequently transmitted to the next generation (Mauch-Mani et al., 2017; Díaz-Valle et al., 2019; Villagómez-Aranda et al., 2022), and research focused on model plants such as Arabidopsis has greatly increased our understanding of the underlying mechanisms (Rasmann et al.; López-Sánchez et al., 2016; Mauch-Mani et al., 2017; Wilkinson et al., 2019; Munekata et al., 2021; Catoni et al., 2022; Wilkinson et al., 2023). Several non-volatile compounds such as isonicotinic acid (INA) and β-aminobutyric acid (BABA) trigger transgenerational immunity (Rasmann et al., 2012; Slaughter et al., 2012; Cohen et al., 2016; Martínez-Aguilar et al., 2016; Buswell et al., 2018). More recent work also provided “Evidence for volatile memory in plants” (Song and Ryu, 2018; Brambilla et al., 2022). Here, we could confirm that transgenerational defence gene priming can be reproduced under variable field conditions: challenging the offspring of nonanal-exposed FJM plants with *C. lindemuthianum* revealed a stronger induction of PR1 and PR4 than in the offspring of control and only challenged plants (**Figure 6**). Thus, our results show that a single exposure to nonanal can be sufficient to trigger an immune response in bean that is associated with improved seed yield in the maternal plants and immunity priming in the offspring generation. This transgenerational effect diversifies the opportunities to use VOCs as ‘crop vaccines’, because technically demanding treatments could be applied by specialized seed-producing companies to provide end-user farmers with ‘vaccinated’, primed seeds.

### 4.4 A pathogen that improves host reproduction

Similarly to nonanal, fungal infection was also associated with higher growth rates and seed yield (**Figures 3**, **9**, **10**). We lack a causal explanation of this phenomenon, although terminal reproductive investment would be an attractive hypothesis. The terminal investment hypothesis predicts higher reproductive investment for individuals that are less likely to survive until future opportunities for reproduction; an effect confirmed for many mammalian and non-mammalian animals (Bonneaud et al., 2004; Duffield et al., 2017). Few studies have tested this hypothesis in plants (Duffield et al., 2017), but root herbivory in mustard (*Sinapis arvensis*) reduced the time that plants needed to enter the flowering stage and enhanced the number of pollinator visits and the numbers of seeds per fruit (Poveda et al., 2003). In our study, the treatments affected the percentage of maternal plants that reached the reproductive stage, and maternal challenging with *C. lindemuthianum* led also to strongly decreased offspring emergence rates, while maternal exposure to nonanal alone enhanced the emergence rate (**Figure 5**). Although the latter result will require confirmation using a larger sample size, all our observations are consistent with the scenario of a “panicking” reproduction at the cost of lower seed quality.

However, the phenomenon of a plant growth promotion by pathogens might be more common than assumed: In general terms, the effects of many plant-colonizing fungi are context dependent and therefore, the net effect might shift along the endophyte-pathogen continuum (Partida-Martinez and Heil, 2011). Moreover, pathogens should actually gain a net fitness benefit from manipulating their host plant to increase growth rate (Heil, 2016). In fact, the volatiles emitted by 11 pathogenic and non-pathogenic root-colonizing fungi promoted plant growth and accelerated flowering, independently of the lifestyle of the fungus (Moisan et al., 2019).

### 4.6 Effects of nonanal on grain quality

Resistance induction reduces the quality of a plant for pathogens or herbivores, and in the end, human consumers are yet another species of herbivore or – in the concrete case of bean - granivore. Therefore, resistance induction can change the taste, nutritional quality or other parameters that affect consumer acceptance. For example, VOCs from mechanically damaged goldenrod plants induced systemic resistance in soybean plants which – although it did not translate to higher seed numbers – reduced the numbers of damaged or infected seeds (Shiojiri et al., 2017, 2020). Unfortunately, the increased resistance in the seeds was partly due to higher contents of isoflavones and saponins of group A (Shiojiri et al., 2017, 2020): two groups of secondary compounds that most strongly contribute to an ‘undesirable’, bitter taste of soybean (Okubo et al., 1992; Yuan et al., 2023). Common bean is an important source of protein and carbohydrates, but it is also appreciated for its high contents of polyphenols. Nonanal exposure during the early vegetative phase indeed affected major classes of polyphenols and antioxidant activity in the harvested grain. While total phenolic compounds showed a strong increase, we found much lower contents of flavonoids and slightly lower contents of condensed tannins in the seeds of nonanal-exposed as compared to control plants (**Table 1**). Still, nonanal exposure slightly increased the antioxidant and radical scavenging activity. These data can be considered – at the very best – as preliminary. Fortunately though, a companion study considered a broader range of techno-functional parameters and reported, e.g., increased protein content and water absorption capacity as well as decreased cooking time for beans from nonanal-exposed plants (Razo-Belmán et al., 2024). The same study also reported increased contents of total phenolic compounds and of flavonoids (Razo-Belmán et al., 2024), hence, results that partly differ from our observations. Future work will have to identify the reason for these contrasting observations. Nevertheless, it seems safe to conclude that nonanal exposure had a positive effect on most and no strong negative effect on some of the parameters that define the functional and nutraceutical quality of beans.

Nonanal exposure alone triggered the production of large seeds with regular shape and homogenous colour; attributes which are very important for consumer acceptance (**Figure 10**). Correspondingly, the participants in our enquiry attributed ‘prime quality’ to these beans. In conclusion (and extrapolating from the very limited characteristics considered here), nonanal can allow for the production of high-quality seeds in the absence of anthracnose, and in case of a subsequent infection of the plants with *C. lindemuthianum*, nonanal exposure at least allows to harvest suitable seeds, i.e. avoids the total loss. Evidently, much more detailed analysis will be necessary to fully evaluate the effects on nonanal exposure on the quality of the harvested grains. Unfortunately, we are not able to compare these findings to other studies, because to the best of our knowledge, besides the work by (Shiojiri et al., 2017, 2020), no study focused on biological resistance induction by VOCs reports quality parameters.

### 4.7 Nonanal exposure can generate net economic benefits in spite of elevated treatment costs

Although roughly 90% of the bean production in México are not sold (Ibarrola-Rivas et al., 2023), beans are also used as cash crop, and the achieved income can help to fulfil important needs. Since many smallholder farmers operate with very limited economic resources, they hardly can afford costly management techniques or commercial products to achieve yield increases. We estimated the costs of our basal management (soil preparation, watering, weeding and fertilization, with no pesticide use) as 9,900 MXN ha^-1^, which is surprisingly close to the 10,794 MXN ha^-1^ calculated by FIRA, a governmental financing agency for the agricultural sector, for the overall production costs of bean (FIRA, 2022). Although this value was calculated for Central Mexico, a cost estimation of ca 110 USD published by the Government of El Salvador (Salvador M.d.A.y.G.d.E, 2020) indicates that these cost are more or less homogeneous over Latin America. However, FIRA allows a total of 1,500 MXN (= ca 90 USD) ha^-1^ season^-1^ for the chemical control of pests, diseases and weeds (FIRA, 2022), while the estimated additional costs of our treatment were ca 7.40 MXN per release point, equivalent to 62,000 MXN ha^-1^. Evidently, these costs can be reduced by re-using the plastic cups and wooden sticks. If cups and stick were re-used, e.g., 5 and 20 times, the increased seed yield of nonanal-exposed FJM plants in trial 1 would have doubled the net profit even when selling to Segalmex (69,000 MXN ha^-1^ net gain achieved with nonanal-exposed plants versus 35,000 MXN ha^-1^ with plants grown under control conditions). and would have generated a 2.2-fold increase in net gain (from 190,000 to 420,000 MXN ha^-1^) if sold via Mercado libre as a high-quality product (**Figure 4**).

In trial 2, the low overall yield would have led to a negative economic balance, if beans were sold at 21,000 MXN t^-1^: this negative balance applies to all five tested cultivars and independently of the re-use of material (**Figure 11**). If sold to a price in the lower range of those found on Mercado Libre, the net economic balance of nonanal exposure would have been positive only for FJM. By contrast, if considering recycling of cups and wooden sticks, nonanal exposure would have created a positive net economic balance for FMA, NSL and FJM beans, which in all three cases was higher than the net gain under control conditions. Assuming, finally, the higher prices of beans sold as ‘premium quality’, ‘organic’ or ‘agroecological’ combined with lowering the treatment costs by re-using material, nonanal exposure of would have generated a net economic profit for four of the cultivars, which in three cases was higher than – and in one case equal to - the net gain under control conditions (**Figure 11**). In addition, we should recall that nonanal exposure of Bayo Berendo beans resulted in an 8-fold higher yield than controls: an increase in the availability of food that might be way more relevant for many smallholder farmers than a theoretical net cost that would apply when selling the beans.

### 4.8 Drawbacks and limitations

Evidently, our study is subject to several drawbacks and limitations that should be considered. For example, trial 2 was performed from August to December, which means in the dry season and so late in the year that several plants (in particular, challenged BB plants) did not even reach the reproductive stage. Still, this situation might simply mimic reality: Smallholder famers may well be forced (or decide for other reasons) to make use of the rainy season to cultivate another, more ‘valuable’ crop and then they have only the dry season to culture bean, which makes this harvest even more important. In this sense, the small - but still significant – yield increases that we observed in the second trial become even more relevant.

A further, more technical limitation of our study is that we did not quantify the concentration of nonanal in the atmosphere, i.e. we do not know to which concentration the plants had been exposed. However, we argue that this information – despite its obvious scientific interest – is perhaps not too relevant in the context of this particular work. First, as stated be others, it is most likely that the concentration of nonanal in the atmosphere at a certain point in time and space differed over various orders of magnitude. Second, and more importantly, similar effects in terms of resistance induction to bacteria were observed in bean plants that had been exposed over 6h or 24h to nonanal at concentrations that differed by an order of magnitude (Girón-Calva et al., 2012), indicating that the concentration at a given time point is not likely to be crucially important. Furthermore, we did not explicitly monitor any potential effects on other species (beneficial or detrimental): nonanal attracts *Spodoptera* females to host plants (Wang et al., 2023) and has been suggested as an active component of the sex pheromones of Asian citrus psyllid, *Diaphorina citri* and greater wax moth, *Galleria mellonella* (Lizana et al., 2023; Luo et al., 2023). However, nonanal was released only once over two days and we did not detect any self-induction of this volatile, two factors that are likely to reduce the potential problems that could resulting from enemy attraction. In the end, effects on any enemy of interest would have shown up as negative effects on yield of seed quality. Finally - and most importantly – the mechanism(s) that underly the observed growth promotion effect and the increased emergence rate of F1 seedlings will have to be deciphered in future studies.

### 4.9 Conclusions and outlook

The medical and the plant sciences share a common problem: the translational gap, a failure to apply scientific knowledge that has been generated under highly controlled experimental to the envisioned system: human beings and cultivated plants. Just like humans, plants face multiple and variable environmental factors when growing under field conditions, which makes it difficult to predict net effects of specific factors from the results of experiments that studied under a very specific and strictly controlled set of conditions the responses of plants to one isolated factor. A very recent study employed aqueous extracts from 11 plant species commonly used as natural pesticides for insect control to reduce fungal anthracnose in bean. In this case, the authors could transfer their findings to ‘real’ smallholder farmers and observed significant yield increases (Kushaha et al., 2024). Similarly, the exposure of rice plants to volatiles emitted from artificially damaged weeds increased yield by 23% and 18%, respectively, in two different years (Shiojiri et al., 2021). The before mentioned studies and the present one show that the “from the field to the lab” approach can work and that testing promising treatments first under variable field conditions might increase the probability of a successful future application “in the real world”.

As mentioned in the Introduction section, genetic variations in the plant immune system and changing environmental factors strongly reduce the reproducibility of the effects of immunity induction across genotypes and, particularly, across experimental conditions. The results obtained with different genotypes of bean confirm the relevance of these factors. As expected, exposure of nonanal reduced the phenotypic disease symptoms in *C. lindemuthianum*-challenged plants of susceptible cultivars like FMA and BaB and the susceptible landrace, NSL, more strongly that in FJM: a cultivar with high basal resistance to anthracnose. Nonanal exposure triggered a statistically significant reduction in numbers of CFUs in the leaves of FJM plants only in the first, but not the second field trial, and quantifying disease symptoms only partly predicted infection rates quantified as CFUs in the second field experiments. A part of these differences can be explained by the high variances that characterize data obtained under natural field conditions. Despite all these issues, stable improvements of plant growth and seed yield could be generated using a single VOC and lanolin paste collocated in commercial plastic cups as dispensers. The transgenerational effect could create an added value to the beneficial effects of immunity priming by nonanal. In addition to the benefits for the environment and the health of the producer, even small-holder farmers could use nonanal to produce and sell vaccinated seeds as an additional source of income. Therefore, we conclude that using volatiles as ‘vaccines’ for plants should be considered a very promising option for biocontrol.

## Data Availability

The raw data supporting the conclusions of this article will be made available by the authors, without undue reservation.
