# Supplementary material for: Immunity priming and biostimulation by airborne nonanal increase yield of field-grown common bean plants
Source: Front Plant Sci. 2024 Nov 5;15:1451864. doi: 10.3389/fpls.2024.1451864 (PMC11577088; doi:10.3389/fpls.2024.1451864)
Supplement: Supplementary file 1 [file Table1.docx]

Immunity priming and biostimulation by airborne nonanal increase yield of field-grown common bean plants

Iris J. Elizarraraz-Martínez^1^, Mariana A. Rojas-Raya^1^, Ana Angélica Feregrino-Pérez^2^, Laila P. Partida-Martínez^3^, & Martin Heil^1*^

^1^Laboratorio de Ecología de Plantas and ^3^Laboratorio de Interacciones Microbianas, Departamento de Ingeniería Genética, CINVESTAV- Unidad Irapuato, Irapuato, México

^2^Facultad de Ingeniería, Universidad Autónoma de Querétaro Campus Amazcala, Querétaro, México

*** Correspondence:** Martin Heil
[martin.heil@cinvestav.mx](mailto:martin.heil@cinvestav.mx)

**Supplementary Text S1**

To estimate the potential economic profit of a yield increase achieved by nonanal, we first calculated the costs of our treatment as a sum of the costs of nonanal (W278203-1KG-K ≥95%, Sigma Aldrich, 1kg: 2.935 MXN), lanolin paste (“lanolina anhidra grado USP” from H6B Oils Centre, purchased via eBay, 1lb: 423 MXN), biodegradable plastic cups (“Vaso Desechable Para Bebidas Calientes”, Bio Form, La Comercial Mexicana: 15 pieces $29.90 MXN, discount for two packages 44.95 MXN) and wooden sticks (purchased in a regular supermarket, 400 MXN per 100 pieces). We calculated the concrete costs per release point (2g lanolin, 20mg nonanal, 1 plastic cup, 1 wooden stick) and extrapolated to a hectare, assuming 8,300 release points ha^-1^ (for details, see Supplementary Table S1). Thereby, we estimated the costs of our treatment as ca. 61,700 MXN ha^-1^, a cost that drops to ca. 19,000 MXN ha^-1^ when the plastic cups and the wooden sticks are re-used 10 and 20 times, respectively (**Supplementary Table S1, Sheet 1**). The basal production costs (tilling, watering and fertilizing), which apply to all four treatments, were estimated as 9,900 MXN per ha and season.

Next, we extrapolated our yield data (which had been obtained in g seed mass plant^-1^) to a hectare, assuming 83,000 plants ha^-1^. To obtain a conservative and an optimistic estimate of the possible income, we assumed that beans are sold to the governmental organism Segalmex (Seguridad Alimentaria Mexicana, <https://www.gob.mx/segalmex>) or, alternatively, as normal or as high -quality (organic) product via the online platform Mercado Libre (<https://www.mercadolibre.com.mx>). Segalmex is a national governmental organism that buys field products at a guaranteed price from rural and small and producers, Mercado Libre is - according to sernagrp.com - the dominating online platform for ecommerce in México that attracts ca 140 million visits per month (Serna, 2022). We used two prices representative of ‘moderate’ and ‘high’ prices (the latter usually for “high quality” or “agro-organic” beans) to calculate the hypothetical value of the yield obtained on one hectare and subtracted the respective cost of the treatmentper hectare to estimate the potential net economic gain.

.

**Table S2 Hypothetical profit achievable with the yield of FJM beans under different treatments**

|  |  | **Sold to Segalmex** | | | | **Sold via Mercado Libre** | | | |
| --- | --- | --- | --- | --- | --- | --- | --- | --- | --- |
|  |  | **C** | **N** | **F*** | **NF** | **C** | **N** | **F*** | **NF** |
| **Recycling**** | **MXN ha^-1^** | 34,500 | 80,100 | 51,300 | 88,500 | 197,400 | 443,000 | 275,600 | 482,500 |
|  | **%** |  | 132.2 | 48.7 | 156.5 |  | 124.4 | 39.6 | 144.4 |
|  | **fold** |  | 2.3 | 1.5 | 2.6 |  | 2.2 | 1.4 | 2.4 |
| **No recycling** | **MXN ha^-1^** | 34,500 | 37,400 | 51,300 | 45,800 | 197,400 | 400,600 | 275,600 | 439,800 |
|  | **%** |  | 8.4 | 48.7 | 32.8 |  | 102.9 | 39.6 | 122.8 |
|  | **fold** |  | 1.1 | 1.5 | 1.3 |  | 2.0 | 1.4 | 2.2 |

Net economic profit estimated as net value of the harvested beans when sold at 21,000 MXN t^-1^to the governmental organisation Segalmex (<https://www.gob.mx/segalmex>) or at a net price of 98 MXN kg^-1^ via the online platform Mercado Libre, minus treatment costs, is indicated in Mexican Pesos (MXN) per hectare rounded to the nearest 100 MXN. For the three treatments, the relative profit increase over the control is indicated in % and as fold change. C, control (sprayed with distilled water); N, nonanal (48 h exposure to nonanal); F, fungus (challenge with *Colletotrichum lindemuthianum*); NF, nonanal exposure followed by challenge with *C. lindemuthianum*). *Economic values of fungus-infected plants are hypothetical because visibly infected seeds cannot be sold. **Recycling refers to the re-use of plastic cups and wooden sticks.

**Table S3 Hypothetical profit achievable with the yield of five bean cultivars subjected to the different treatments**

|  |  | **No recycling** | | | | |  | **Recycling*** | | |  |
| --- | --- | --- | --- | --- | --- | --- | --- | --- | --- | --- | --- |
|  |  | **C** | **N** | **F**** | **NF** |  | | **C** | **N** | **NF** |  |
| **Segalmex** | FMA | -5,600 | -48,300 | -4,100 | -42,600 |  | | -5,600 | -5,600 | 100 |  |
|  | PV | -3,600 | -62,400 | -4,400 | -60,500 |  | | -3,600 | -20,800 | -18,800 |  |
|  | NSL | -7,100 | -55,500 | -1000 | -56,500 |  | | -7,100 | -13,900 | -14,800 |  |
|  | FJM | -5,100 | -54,600 | -100 | -55,000 |  | | -5,100 | -13,000 | -13,400 |  |
|  | BB | -8,300 | -66,800 | -9,900 | -71,800 |  | | -8,300 | -25,200 | -30,200 |  |
| **Low** | FMA | 2,300 | -34,300 | 6,600 | -18,500 |  | | 2,300 | 7,400 | 23,100 |  |
| **online** | PV | 4,300 | -50,800 | 2,500 | -46,400 |  | | 4,300 | -9,100 | -4,800 |  |
|  | NSL | -3,300 | -32,900 | 11,300 | -35,300 |  | | -3,300 | 8,700 | 6,300 |  |
|  | FJM | 10,300 | 200 | 31,100 | -1,400 |  | | 10,300 | 41,800 | 40,200 |  |
|  | BB | -2,700 | -49,000 | -9,900 | -71,800 |  | | -2,700 | -7,400 | -30,200 |  |
| **High** | FMA | 20,600 | 21,900 | 31,400 | 61,500 |  | | 20,600 | 63,500 | 103,200 |  |
| **online** | PV | 27,800 | -15,800 | 23,000 | -4,200 |  | | 27,800 | 25,800 | 37,400 |  |
|  | NSL | 5,400 | 17,600 | 38,900 | 12,200 |  | | 5,400 | 59,300 | 53,800 |  |
|  | FJM | 12,600 | 8,400 | 35,700 | 6,600 |  | | 12,600 | 45,000 | 48,000 |  |
|  | BB | -2,500 | -48,300 | -9,900 | -71,800 |  | | -2,500 | -6,700 | -30,200 |  |

The net economic profit of five bean cultivars was estimated assuming that beans are sold to the governmental organism Segalmex (Seguridad Alimentaria Mexicana, <https://www.gob.mx/segalmex>) or, alternatively, as normal or as high -quality (organic) product via the online platform Mercado Libre, minus treatment costs, and are indicated in Mexican Pesos (MXN) per hectare rounded to the nearest 100 MXN. C, control (sprayed with distilled water); N, nonanal (48 h exposure to nonanal); F, fungus (challenge with *Colletotrichum lindemuthianum*); NF, nonanal exposure followed by challenge with *C. lindemuthianum*). *Recycling refers to the re-use of plastic cups and wooden sticks. **Economic values of fungus-infected plants are hypothetical because visibly infected seeds cannot be sold. They are presented only once since they do not differ depending on recycling.
